# Enrichment characteristics and dietary evaluation of selenium in navel orange fruit from the largest navel orange-producing area in China (southern Jiangxi)

**DOI:** 10.3389/fpls.2022.881098

**Published:** 2022-08-08

**Authors:** Fengxian Yao, Li Wen, Rong Chen, Chao Du, Shiming Su, Mengmeng Yan, Zhonglan Yang

**Affiliations:** ^1^National Navel Orange Engineering Research Center, School of Life Sciences, Gannan Normal University, Ganzhou, China; ^2^School of Geography and Environmental Engineering, Gannan Normal University, Ganzhou, China; ^3^Jiangxi Provincial Key Laboratory for Low-Carbon Recycling Technology of Municipal Solid Waste, Ganzhou, China; ^4^Institute of Environment and Sustainable Development in Agriculture, Chinese Academy of Agricultural Sciences, Beijing, China

**Keywords:** selenium enrichment, navel orange, elemental coupling, dietary evaluation, selenium intake

## Abstract

Diet is the main intake source of selenium (Se) in the body. Southern Jiangxi is the largest navel orange-producing area in China, and 25.98% of its arable land is Se-rich. However, studies on the Se-rich characteristics and Se dietary evaluation of navel orange fruits in the natural environment of southern Jiangxi have not been reported. This study was large-scale and *in situ* samplings (*n* = 492) of navel oranges in southern Jiangxi with the goal of investigating the coupling relationships among Se, nutritional elements, and quality indicators in fruits and systematically evaluating Se dietary nutrition to the body. The results indicated that the average content of total Se in the flesh was 4.92 μg⋅kg^–1^, and the percentage of Se-rich navel oranges (total Se ≥ 10 μg⋅kg^–1^ in the flesh) was 7.93%, of which 66.74% of the total Se was distributed in the pericarp and 33.26% in the flesh. The average content of total Se in the flesh of Yudu County was the highest at 5.71 μg⋅kg^–1^. There was a significant negative correlation (*p* < 0.05) between Se, Cu, and Zn in the Se-rich flesh. According to the Se content in the flesh, the Se dietary nutrition evaluation was carried out, and it was found that the Se-enriched navel orange provided a stronger Se nutritional potential for the human body. These findings will help to identify Se enrichment in navel orange fruit in China’s largest navel orange-producing area and guide the selection of Se-rich soils for navel orange production in the future.

## Graphical Abstract

Distribution characteristics and dietary evaluation of Se-enriched navel oranges in southern Jiangxi, China. 
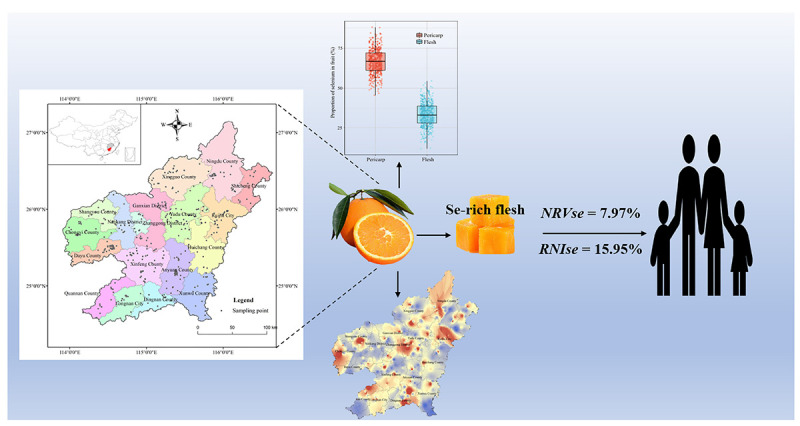

## Introduction

Selenium (Se) is a metalloid element found in nature and is an essential trace element in the human body (Bañuelos et al., 2016; Mao et al., 2016; Kieliszek, 2019). Diet is the main intake source of body Se, and excessive (>400 μg⋅d^–1^) or insufficient (<50 μg⋅d^–1^) intake of Se in humans (ages ≥ 18 years) can cause discomfort and disease (National Health Commission of the People’s Republic of China, 2017). Globally, Se deficiency is more prevalent than Se poisoning and is of wider concern (Xiao et al., 2020). Se deficiency can easily result in a decrease in human immunity and can threaten human health (Kieliszek et al., 2017; Kieliszek, 2019; Motesharezadeh et al., 2021). Some endemic diseases, such as Keshan disease, have been demonstrated to be closely related to Se deficiency in the ecological environment (Wang and Gao, 2001; Shi et al., 2017). According to the WHO report, China is one of the 40 Se-deficient countries in the world (Xu et al., 2010; Li et al., 2015; Dinh et al., 2018). It was reported that approximately 51% of China’s land is Se deficient, and 39–61% of the Chinese population has a daily Se intake of approximately 26–34 μg, which is lower than the WHO/FAO recommended minimum intake of 50 μg⋅d^–1^ Se (WHO/FAO, 1996; Dinh et al., 2018; Zhai et al., 2021). In the Keshan disease areas, the daily Se intake is even lower than 17 μg (Ge and Yang, 1993; Zhai et al., 2021). Thus, it is important to investigate the Se richness of representative crop (fruits)-producing areas in China.

Navel oranges, a type of citrus, are one of the most popular orange varieties and are now widely grown in 114 tropical and subtropical countries, including China, Brazil, and the United States (Liu et al., 2013; Chen et al., 2015). Southern Jiangxi is the largest navel orange production area in China, with an area of 1,100 km^2^ and a total annual production of 1.2 million tons (Yang et al., 2017). Navel oranges with total Se content ≥ 10 μg⋅kg^–1^ in the edible part are called Se-rich navel oranges (All China Federation of Supply and Marketing Cooperatives, 2017; Dinh et al., 2018). The enrichment of Se in navel orange fruits is strongly influenced by the background content of soil Se (Lyu et al., 2021). Natural soils with background Se contents greater than 0.4 mg⋅kg^–1^ are called Se-rich soils (Dinh et al., 2018). According to the results of soil data from the 1:50,000 land quality geochemical survey in southern Jiangxi (China Geological Survey, 2019), the background Se content in arable soils in southern Jiangxi was greater than 0.4 mg⋅kg^–1^ in an area spanning 4114.51 km^2^, accounting for 25.98% of the total arable land area (Supplementary Table 1). The enrichment of Se in edible parts of crops or fruits is also influenced by the coupling of a number of nutrients (Natasha et al., 2018). There are issues that need to be studied in the edible portion of navel oranges, including the enrichment characteristics of Se, the coupling relationship between Se and nutrient elements, and the effect of Se on human dietary nutrition. However, there are few reports on the above issues in China’s largest navel orange producing area.

In this study, 18 counties in southern Jiangxi were selected for large-scale and *in situ* sampling of navel orange fruit, focusing on Se in the edible part of navel oranges. The objectives of this work are (1) to explore the enrichment characteristics and spatial distribution patterns of Se in navel orange fruit; (2) to investigate the coupling relationship between Se and nutrients in fruit; and (3) to evaluate the dietary nutrition of Se to the body based on Se content in flesh. This work will help refine the dietary profile of Se in China, especially *via* the navel orange intake channel, as well as optimise the layout of Se-rich navel oranges in China’s largest navel orange-producing area.

## Materials and methods

### Study area

The study area is located in southern Jiangxi Province, China, with a geographical area of 24°29′ N – 27°09′ N, 113°54′ E – 116°38′ E, a land area of 39,379.64 km^2^, and a resident population of 8,780,000. Located in the subtropics, it has a typical subtropical monsoonal humid climate with an annual average temperature of 18.9^°^C, an annual average frost-free period of 287 d, an annual average rainfall of 1,605 mm, annual sunshine hours of 1,813 h, and a large temperature difference between day and night, which makes it very suitable for the growth of navel oranges. According to the previous survey results of Jiangxi Soil and Fertiliser Technical Extension Station, the soil in southern Jiangxi is a typical kanhapludults (the American soil system classification), with an average pH value of 5.2 ± 0.40, an average organic matter value of 28.5 ± 9.22 g⋅kg^–1^, an average alkali-hydro nitrogen value of 144.4 ± 51.78 mg⋅kg^–1^, an average rapidly available phosphorus value of 21.8 ± 14.54 mg⋅kg^–1^, and an average rapidly available potassium value of 74.5 ± 42.57 mg⋅kg^–1^. Details of the total Se content and distribution area in the arable soil are shown in Supplementary Table 1.

### Sample collection

**FIGURE 1:**
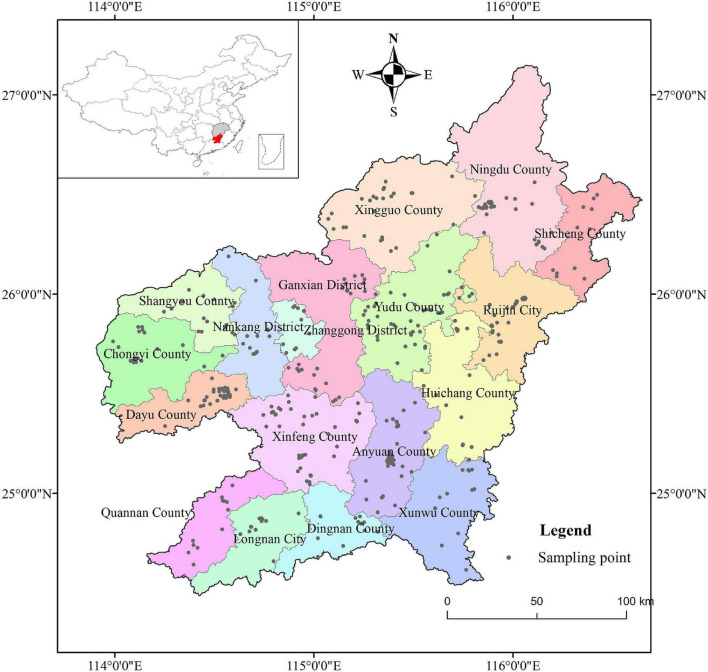
Locations of the study area and 492 fruit sampling points in southern Jiangxi Province, China.

A total of 492 navel orange fruit samples were collected from 18 counties, cities, or districts in southern Jiangxi, China, between October and November 2019. The latitude and longitude of the collected samples are detailed in Supplementary Table 2, and the distribution of collection sites is shown in Figure 1. Care was taken to make sure the samples had similar size, bright colour, no obvious damage, insect infestation and other trauma, and to maintain the integrity of the samples. The samples collected were all Newhall navel oranges. After collection, the samples were labelled and recorded and stored in fresh bags for preservation. The follow-up sample processing and determination analysis were performed at the National Navel Orange Engineering Research Center of Gannan Normal University. All chemicals used in this work were of analytical grade and were purchased from Beijing Chemical Reagents Co. (Beijing, China).

### Analysis of general indicators of navel orange fruit quality

After the fruit samples were processed, the single fruit weight and the fruit horizontal diameter (H), longitudinal diameter (D), and pericarp thickness were determined using an electronic scale (AL204, Mettler-Toledo Instruments Ltd., Switzerland) and a Vernier caliper (0.02 mm), respectively; the longitudinal diameter is measured from the bottom to the top of the fruit, the horizontal diameter is measured at the equatorial surface of the fruit, and the fruit shape index = H/D is then calculated. The soluble solids content of the juice solution was determined with a portable refractometer (Refracto30GS, Mettler-Toledo Instruments Ltd., Switzerland), the vitamin C content was determined by titration with 2,6-dichloroindophenol, and the titratable acid content was determined by titration with a 0.1 mol⋅L^–1^ NaOH solution (Ruck, 1961; Khan et al., 2020).

### Determination of selenium in the flesh and pericarp of navel oranges

After the separation of navel orange flesh and pericarp, Se determination analysis was carried out according to the method provided by Xiao et al. (2020) and Lyu et al. (2021). A brief description is as follows: A solid sample of 0.5 g was added to 10 mL of HNO_3_-HClO_4_ (v/v = 4/1) buffer and left to stand overnight at room temperature; it was then preheated at 60°C for 30 min, to remove the white smoke, and then heated continuously at 160°C until the volume of the solution was less than 1 mL. After dilution and volume determination, the solution was obtained, and the Se concentration was measured using hydride-atomic fluorescence spectrometry (HG-AFS 9120, Beijing Jitian Instruments Co., Ltd., China) with a detection limit of 0.001 mg⋅L^–1^. The recovery of Se was 94.2 ± 2.8% using a standard substance of wheat grain (GBW-10011) for quality control. The total Se content of the fruit was obtained based on the Se content and mass contribution of pericarp and flesh.

### Determination of mineral elements in the flesh and pericarp of navel oranges

The main elements to be measured are the massive elements P and K, the medium elements Ca and Mg, and the trace elements Fe, B, Mn, Cu, Zn, and Mo. The above elements were determined according to the method provided by Barros et al. (2012) and Czech et al. (2020), which is briefly described as follows: The finely ground samples were oven dried at 60 °C and accurately weighed to 0.3 g in a crucible; the samples were then placed in a muffle furnace, ashed at 150°C and 300°C for 1 h each, and then ashed at 500°C for 5 h; the result was an off-white ash. The samples were cooled to room temperature on an asbestos sheet; then, 10 mL of 1 mol⋅L^–1^ HCl was added, allowed to react, and the solution was filtered to determine B, Mn, Fe, Cu, Zn, Mo, Mg, P, K, and Ca using an inductively coupled plasma mass spectrometer (ICP–MS, Agilent 7900, United States). The mineral element content of the fruit was obtained based on the mineral element content and mass contribution of the pericarp and flesh of the fruit.

### Dietary nutritional evaluation of selenium in navel oranges

Dietary nutrition evaluation of Se mainly uses the nutrient reference value (*NRV*), which is the amount of nutrients per unit of food as a percentage of the daily nutrient requirement or recommended intake (National Health and Medical Research Council, 2006; Chinese Nutrition Society, 2010; Stamm and Houghton, 2013). The recommended nutrient intake (*RNI*) is the percentage of the recommended intake of a nutrient that is required for 97–98% of the dietary requirements of a given group of individuals (specific gender, age and individual physical health status) (National Health and Medical Research Council, 2006; Chinese Nutrition Society, 2010; Stamm and Houghton, 2013). Unlike NRV, which focuses on the nutrient intake of the general population, RNI is more targeted and can evaluate the nutritional status of different sex and age groups. The obtained values of Se content of navel orange flesh in southern Jiangxi were substituted into Equations (1) and (2) to calculate the *NRVse* (%) and *RNIse* (%), respectively, of Se per 100 g of navel orange fruit.


(1) $$NRVse\left(\%\right)=\frac{C}{10\times N\,R\,V}\times 100\%$$

(2) $$RNIse\left(\%\right)=\frac{C}{10\times R\,N\,I}\times 100\%$$

where *C* is the Se content of navel orange flesh (μg⋅kg^–1^), *NRV* is the nutrient reference value for Se, 50 μg (Chinese Nutrition Society, 2016), and *RNI* is the recommended daily dietary intake for Se (μg) (Supplementary Table 3).

### Statistical analysis

Statistical analyses were performed using SPSS 20.0 software (IBM Corporation, Chicago, IL, United States). The spatial distribution of Se in navel orange fruit and flesh was obtained by kriging interpolation, a geostatistical method performed using ArcGIS software (ArcGIS10.2, ESRI, RedLands, CA, United States). Origin 2018 software (OriginLab Inc., Northampton, Massachusetts, United States) and the R programming language^1^ were used for plotting graphs. All reported significant differences are at the *p* < 0.05 level unless otherwise stated.

## Results

### Total selenium in navel orange fruit

**FIGURE 2:**
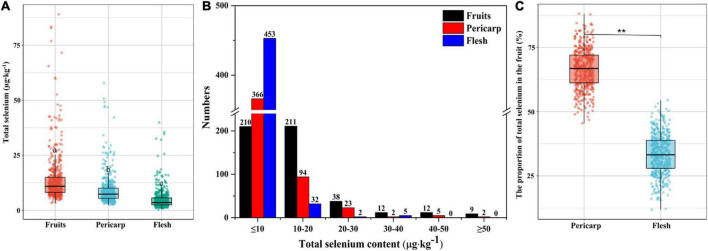
Total Se content of navel orange fruit, pericarp, and flesh in southern Jiangxi **(A)**, distribution of total Se content in fruit **(B)**, and percentage of total Se in flesh and pericarp (*n* = 492) **(C)**. Letters indicate a significant difference at *p* < 0.05 among treatments (**p < 0.01).

The distribution of total Se in the fruit, pericarp, and flesh of navel oranges in southern Jiangxi is shown in Figure 2. The average content of total Se in fruit, pericarp, and flesh was 14.19 ± 11.13, 9.27 ± 7.03, and 4.92 ± 4.17μg⋅kg^–1^, respectively. The proportion of total Se content < 10 μg⋅kg^–1^ in the pericarp and flesh of navel oranges were 74.39 and 92.07%, respectively, while those of ≥ 10 μg⋅kg^–1^ were 25.61 and 7.93%, respectively (Figure 2B and Supplementary Table 4). Further analysis revealed that an average of 66.74% of the total Se in the fruit was distributed in the pericarp and 33.26% in the flesh (Figure 2C).

### Spatial distribution of total selenium in the fruit and flesh of navel oranges

**FIGURE 3:**
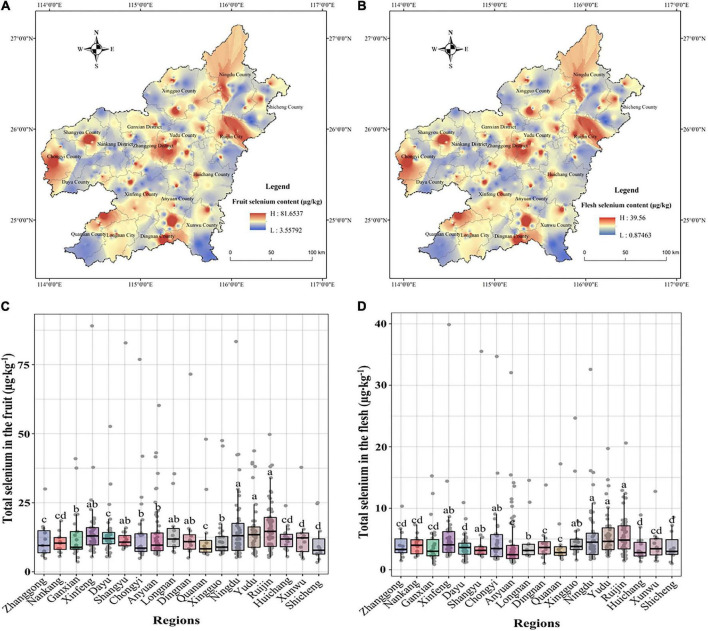
Total Se content and spatial distribution in navel orange fruit **(A,C)** and flesh **(B,D)** in southern Jiangxi (*n* = 492). Letters indicate a significant difference at *p* < 0.05 among treatments.

The spatial distribution of total Se in navel orange fruit and flesh in southern Jiangxi was shown in Figures 3A,B, and the variation in total Se content was shown in Figures 3C,D. The distribution in the 18 counties sampled showed the following regularity: The uneven distribution of total Se in the fruit and flesh was clearly reflected by the presence of multiple Se-rich centre points, which spread outwards in a radial pattern (Figures 3A,B). The spatial distribution pattern of total Se in the flesh is of greater interest, as shown by Supplementary Table 6. The average content of total Se in flesh was higher in Yudu (5.71 μg⋅kg^–1^), Ruijin (5.67 μg⋅kg^–1^), and Ningdu (5.49 μg⋅kg^–1^) counties and lower in Huichang (3.55 μg⋅kg^–1^), Dayu (3.77 μg⋅kg^–1^), and Shicheng (3.80 μg⋅kg^–1^) counties (Supplementary Table 5).

### Fruit quality indicators and mineral elements of navel oranges

Quality indicators and mineral elements of ordinary navel oranges (Se < 10 μg⋅kg^–1^ in the flesh) and Se-rich navel oranges (Se ≥ 10 μg⋅kg^–1^ in the flesh) in southern Jiangxi were shown in Table 1. The distribution of different mineral elements in ordinary and Se-rich navel oranges showed the following regularity: The average content of trace elements (B, Fe, Mn, Cu, Zn, and Mo) in ordinary navel oranges was not significantly different from that in Se-rich navel oranges (p > 0.05), and the average content in Se-rich flesh was 16.10 ± 3.57 (B), 5.29 ± 3.79 (Mn), 12.72 ± 2.12 (Fe), 2.58 ± 0.64 (Cu), 3.78 ± 0.82 (Zn), and 0.028 ± 0.014 (Mo) mg⋅kg^–1^, respectively (Table 1 and Supplementary Table 2). However, the average content of medium and massive elements (Mg, P, K, and Ca) was higher in Se-rich navel oranges than in ordinary navel oranges; specifically, the average values were 91.04 ± 14.22 (Mg), 130.17 ± 33.07 (P), 1,141 ± 194 (K), and 244 ± 59.01 (Ca) mg⋅kg^–1^ in Se-rich flesh (Table 1 and Supplementary Table 2). Among these elements, P and K differed from other elements in that they are mainly distributed in the flesh, where they account for 70.81–72.14% and 57.48–57.53%, respectively (Table 1 and Supplementary Table 2).

**TABLE 1:** Quality indicators and mineral elements of ordinary navel orange (total Se < 10 μg⋅kg^–1^ in the flesh) and Se-rich navel orange (total Se ≥ 10 μg⋅kg^–1^ in the flesh) fruit (**p < 0.01).

Indicators		Ordinary navel oranges (Mean ± *SD*, *n* = 453)	Se-rich navel oranges (Mean ± *SD*, *n* = 39)
Single fruit weight (g)		234.52 ± 43.82	251.84 ± 24.52
Horizontal diameter (H) (mm)		76.08 ± 5.07	78.82 ± 3.28
Longitudinal diameter (D) (mm)		78.64 ± 5.37	81.11 ± 3.79
H/D		0.968 ± 0.033	0.973 ± 0.033
Pericarp thickness (mm)		4.65 ± 0.77	4.82 ± 0.88
Soluble solids (%)		12.03 ± 1.33	11.89 ± 1.17
Vitamin C (mg⋅100 g^–1^)		52.07 ± 7.36	52.28 ± 8.09
Titratable acid (%)		0.719 ± 0.218	0.718 ± 0.159
Se (μg⋅kg^–1^)	Fruit	11.73 ± 5.19	43.82 ± 16.05**
	Flesh	3.83 ± 1.89	16.60 ± 7.69**
B (mg⋅kg^–1^)	Fruit	40.79 ± 5.25	40.75 ± 4.90
	Flesh	16.12 ± 3.52	16.10 ± 3.57
Mn (mg⋅kg^–1^)	Fruit	21.85 ± 13.10	21.46 ± 11.71
	Flesh	5.26 ± 4.25	5.29 ± 3.79
Fe (mg⋅kg^–1^)	Fruit	31.66 ± 23.77	30.72 ± 7.67
	Flesh	12.71 ± 4.86	12.72 ± 2.12
Cu (mg⋅kg^–1^)	Fruit	6.20 ± 2.69	6.18 ± 2.19
	Flesh	2.61 ± 0.63	2.58 ± 0.64
Zn (mg⋅kg^–1^)	Fruit	10.01 ± 3.04	9.93 ± 2.59
	Flesh	3.81 ± 0.88	3.78 ± 0.82
Mo (mg⋅kg^–1^)	Fruit	0.064 ± 0.048	0.063 ± 0.046
	Flesh	0.025 ± 0.018	0.028 ± 0.014
Mg (mg⋅kg^–1^)	Fruit	188.09 ± 42.64	189.89 ± 23.88
	Flesh	89.88 ± 14.88	91.04 ± 14.22
P (mg⋅kg^–1^)	Fruit	176.83 ± 37.96	180.43 ± 33.72
	Flesh	125.22 ± 29.29	130.17 ± 33.07
K (mg⋅kg^–1^)	Fruit	1,940 ± 284	1,985 ± 314
	Flesh	1,116 ± 180	1,141 ± 194
Ca (mg⋅kg^–1^)	Fruit	901 ± 214	907 ± 218
	Flesh	237 ± 70.61	244 ± 59.01

### Correlation of navel orange fruit quality indicators, mineral elements, and selenium

**FIGURE 4:**
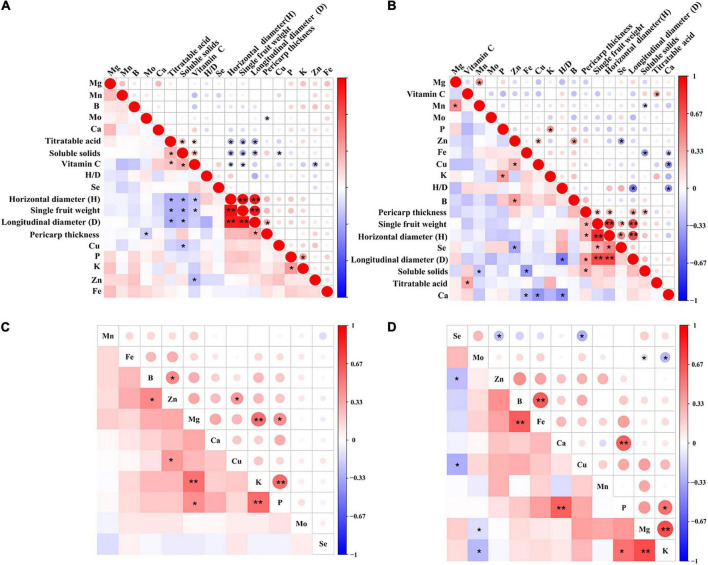
Correlation analysis of Se with its quality indicators and mineral elements in ordinary navel orange fruit **(A)** and flesh **(C)** and Se-rich navel orange fruit **(B)** and flesh **(D)** in southern Jiangxi (*p < 0.05, **p < 0.01).

The Pearson correlation coefficient among Se and all indicators in ordinary navel orange fruit from southern Jiangxi showed no significant correlation (*p* > 0.05) (Figure 4A). In contrast, a significant negative correlation was found between Se and Zn in the Se-rich fruit, and a significant positive correlation (*p* < 0.05) was found with quality indicators such as single fruit weight and horizontal diameter (H) (Figure 4B). No significant relationship was found between Se and all mineral elements in the flesh of ordinary navel oranges (*p* > 0.05) (Figure 4C); potentially weak positive correlations were found with Fe, Cu, and Mo, and weak negative correlations were found with other elements (p > 0.05); in addition, significant positive correlations were found among P, K, and Mg elements (*p* < 0.05). In the Se-rich flesh (Figure 4D), a significant negative correlation was found between Se, Cu, and Zn (*p* < 0.05); a non-significant weak positive correlation was found between Mo, Mg, and K (*p* > 0.05); and a significant coupled positive correlation was found between P, K, and Mg elements (*p* < 0.05). The above analysis indicates that Se in the flesh of Se-rich navel oranges may be influenced by the coupling of elements such as Cu and Zn. Other mineral elements, although not directly and significantly influencing Se enrichment, may indirectly influence Se enrichment through the coupling between elements, especially P, K, and Mg.

### Nutritional evaluation of selenium diets based on selenium content of navel orange flesh

**FIGURE 5:**
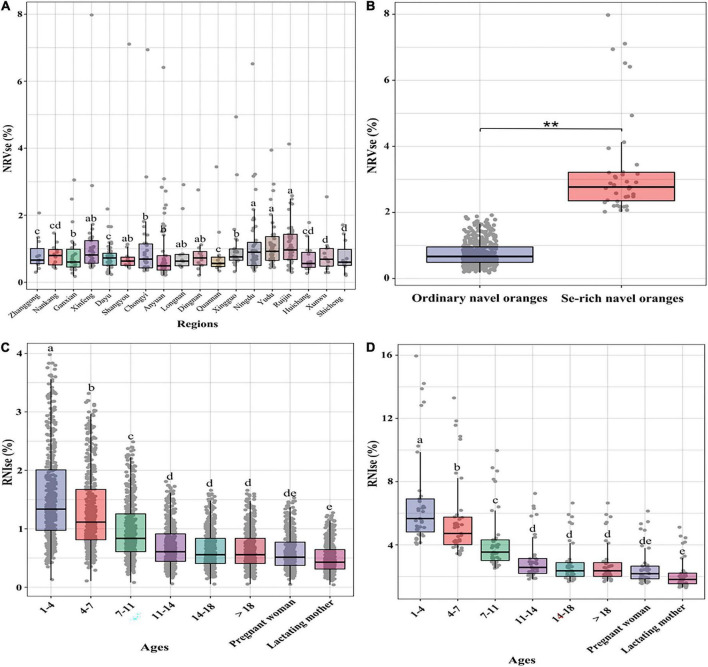
The difference in NRVse in the study area **(A)**, the difference in NRVse between ordinary and Se-rich navel oranges **(B)**, and the difference in RNIse between ordinary **(C)** and Se-rich **(D)** navel oranges. Letters indicate a significant difference at *p* < 0.05 among treatments (**p < 0.01).

The Se dietary nutrition evaluation was based on the Se content of navel orange flesh in southern Jiangxi and mainly used *NRVse* and *RNIse*. Among the study areas, the average value of *NRVse* is the highest in Yudu County (1.142%), followed by Ruijin city (1.134%), Ningdu County (1.099%), Xingguo County (1.077%), and Xinfeng County (1.061%); Shicheng, Dayu, and Huichang counties were lower at 0.761, 0.754, and 0.711%, respectively (Figure 5A). The average value of *NRVse* in ordinary navel oranges is only 0.767%, while the *NRVse* of Se-rich navel orange flesh can reach 3.320% (Figure 5B). This means that Se-rich navel oranges can meet the body’s nutritional requirements better than ordinary navel oranges; this is especially true of oranges produced in Yudu, Ruijin, and Ningdu, which can function as natural dietary Se supplements.

The values of *RNIse* varied in the study area, and as with *NRVse*, the highest average values of *RNIse* were found in Yudu County, with values of 2.854% for ages 1–4, 2.283% for ages 4–7, 1.631% for ages 7–11, 1.268% for ages 11–14, and 1.142% for ages > 14; the values of the special groups of pregnant women and lactating mothers were 1.142 and 0.878%, respectively (Supplementary Figure 1). The *RNIse* differences between ordinary and Se-rich navel oranges were analysed further. The average value of *RNIse* in ordinary navel oranges is only 1.233% (Figure 5C), while the *RNIse* of Se-rich navel orange flesh can reach 5.338% (Figure 5D). A gradual increase in *RNIse* values with age was observed, with Se-rich navel oranges having an *RNIse* value approximately 4.33 times higher than that of ordinary navel oranges.

## Discussion

### Bioavailability and transport mechanisms of selenium in navel oranges

Se accumulation in navel orange fruit is mainly dependent on the Se content in the soil (Sieprawska et al., 2015; Lyu et al., 2021). The bioavailability of Se is mainly influenced by the chemical form of Se, soil pH, organic matter, redox potential (Eh), competing ions, and microbial activity (Sieprawska et al., 2015; Natasha et al., 2018). Se(VI) is predominant at alkaline pH, while at acidic and neutral pH, Se is mainly present as selenite [Se(IV)] (Zhu et al., 2009; Bañuelos et al., 2016). Soil organic matter (SOM) also plays a key role in regulating the mobility and bioavailability of Se (Tolu et al., 2014; Natasha et al., 2018). SOM has a high binding affinity for Se due to the presence of functional groups such as phenols, alcohols, and quinones (Niazi et al., 2015; Natasha et al., 2018), especially in acidic soils (Li et al., 2017; Natasha et al., 2018). In this study, the background Se content of soils in the study area to some extent determined the spatial variability of Se in navel orange flesh (Supplementary Table 1) (for instance, in Yudu and Shicheng counties), but key soil physicochemical factors such as pH and SOM also strongly modulated the bioavailability of Se. According to available research data, the average values of pH and SOM of soils in southern Jiangxi were 5.2 ± 0.40 and 28.5 ± 9.22 g⋅kg^–1^, respectively. Regional variation in these indicators is also an important factor contributing to the spatial variation in Se in southern Jiangxi navel orange fruit; however, the underlying mechanisms in this process need to be explored in more depth in the future.

There is no doubt that the bioavailability of Se is ultimately achieved through soil–plant transfer channels and processes, the intrinsic mechanisms of which, although already systematically discussed (White, 2016, 2018; Natasha et al., 2018; Zhao et al., 2020), need to be described once again in this paper. A brief summary is as follows: Plants cannot take up elemental Se directly because these forms of Se are insoluble (Fernández-Martínez and Charlet, 2009; Yuan et al., 2022). Plants have a greater affinity for Se(VI) than Se(IV) in the soil (Tian et al., 2016). Se is chemically similar to S, and Se(VI) enters the plant mainly *via* sulphate transporters (SULTR1;1/1;2) on the plasma membrane of root cells (Bitterli et al., 2010; Schiavon et al., 2016). Se(IV) may be transported *via* phosphate (P) transporters, such as OsPT2, a P transporter, which has been reported to be involved in the active uptake of Se(IV) by plant roots (Zhang L. H. et al., 2014). Studies have demonstrated that more than half of the Se(VI) applied to plant roots is transferred to the overground (shoots), while Se(IV) remains in the root tissue (Arvy, 1993). Se(VI) can be transferred from the roots to the overground (shoots) *via* the xylem and then *via* the xylem and phloem to the fruit and seeds (reproductive organs) (Zhu et al., 2009; Natasha et al., 2018). Members of the ALMT family, such as AtALMT12, can load Se(VI) into xylem sap, and Se(VI) is the predominant Se form in the xylem, with small amounts of selenomethionine (SeMet) and methylselenocysteine (MeSeCys) also present (White, 2018; Zhao et al., 2020). Organic Se, such as selenocysteine (SeCys), SeMet, and MeSeCys, likely enter plant cells *via* amino acid transporters (AATr) and the NRT1;1B transporter (Zhang et al., 2019; Schiavon et al., 2020; Zhao et al., 2020). Overexpression of the NRT1;1B transporter enhanced the transfer of SeMet to seeds (Zhang et al., 2019). SeMet can also be methylated by methionine methyltransferase and converted to methyl SeMet, which in turn is methylated to dimethyl selenide (DMSe), a non-toxic compound of Se which is volatile in the atmosphere (Gupta and Gupta, 2017; Natasha et al., 2018).

### Relationships among selenium, mineral elements, and quality indicators in navel oranges

In this study, the enrichment of Se in the fruit significantly contributed to the improvement of fruit quality, including fruit weight and horizontal diameter (Figure 4B). Meanwhile, the fruit shape index (H/D) and vitamin C content of Se-rich navel oranges were higher than those of ordinary navel oranges, but the titratable acid content was lower than that of ordinary navel oranges (Table 1). This is consistent with the findings of Wen et al. (2021), who demonstrated that exogenous application of Se fertiliser (150 mg⋅L^–1^ Se) significantly promoted citrus growth and development, increasing single fruit weight, horizontal and longitudinal diameter, and vitamin C content, while reducing acidity (*p* < 0.05). This is mainly attributed to the fact that the application of Se fertiliser promotes an increase in chlorophyll content in the plant body and promotes photosynthesis (Zhang M. et al., 2014; Wen et al., 2021). The assimilates of photosynthesis are the material basis of plant cell activity, and the accumulation of photosynthates directly affects a range of metabolic activities (Matsoukas et al., 2012; Wen et al., 2021). Meanwhile, Se fertilisation application significantly promoted the activity of antioxidant enzymes in leaves and fruits, facilitated the absorption of nutrients (Motesharezadeh et al., 2019), increased vitamin C content, reduced acidity, promoted the accumulation of total Se content in fruits, and improved fruit quality (Ardebili et al., 2014; Wen et al., 2021).

In this study, the average content of medium and massive elements (Mg, P, K, and Ca) was higher in Se-rich navel oranges than in ordinary navel oranges (Table 1), indicating that Se can promote the co-uptake of the above elements (Bañuelos et al., 2020). This is consistent with the findings of Wen et al. (2021), and this phenomenon has been confirmed in other crops, such as tea and pear trees (Liu et al., 2015; Qin et al., 2019). Meanwhile, this study found a significant coupled positive correlation (*p* < 0.05) between P, K, and Mg elements in Se-rich navel oranges (Figure 4). This phenomenon may be attributed to the fact that P, K, and Mg are essential nutrients for fruit and are involved in key metabolic activities, such as photosynthesis and carbohydrate synthesis, and large selective uptake improves fruit quality (Lambers et al., 1998). In this study, a significant negative correlation (*p* < 0.05) was found between Se, Cu, and Zn in the flesh of Se-rich navel oranges (Figure 4D). It was reported that excessive accumulation of Cu inhibits chloroplast enzyme activity and affects photosynthesis (Huo et al., 2020), while the opposite is true for Se. Cu is mostly present in the soil as Cu^2+^-O-Fe^3+^ and Cu-O-Al (Luo et al., 2003), inhibiting the bioavailability of Cu. Hu et al. (2011) reported that the ability of pakchoi to enrich Se was much greater than the ability of pakchoi to enrich Cu in co-contaminated soils. Se and Zn are both fourth-period elements with similar atomic numbers and may be similar and competitive at the molecular level (He et al., 2004). It was reported that Se and Zn application in combination affects the uptake and translocation of Se and Zn by plants (Mangueze et al., 2018; Motesharezadeh et al., 2020). Xue et al. (2020) reported that pakchoi enriched more Se than Zn in co-contaminated soils. In this study, an interesting phenomenon was found in that P and K elements, unlike other elements, were predominantly distributed in the flesh, where they accounted for 70.81–72.14% and 57.48–57.53% of total content, respectively (Supplementary Table 1). This is consistent with the findings of Xie et al. (2012). This may be because the fruit samples were collected at fructescence (October–November), when P and K are involved in the synthesis of fruit carbohydrates (Lambers et al., 1998), resulting in a significant enrichment in the flesh.

### Nutrient reference value and recommended nutrient intake evaluation of selenium

Based on the Se content of navel orange flesh in southern Jiangxi, this study theoretically evaluated the dietary nutritional contribution of Se to the body. The highest values of Se nutrient reference values (*NRVse*) and recommended dietary intake (*RNIse*) assessed were 7.97 and 15.95%, respectively. This suggests that Se deficiency is a primary concern in future diets. Considering the key role that Se plays in human health, this work is indispensable for safeguarding human health. Overall, understanding the enrichment characteristics and spatial distribution pattern of Se in the edible part of navel oranges in China’s largest navel orange-producing area and conducting dietary evaluation could help refine the dietary profile of Se in China, especially *via* the navel orange channel.

## Conclusion

The background content of soil Se determines the spatial distribution of Se in the edible part of navel oranges in the natural environment. Approximately 67% of the total Se is distributed in the pericarp and 33% is distributed in the flesh. Se-rich navel oranges accounted for 7.93% of the survey volumes. The enrichment of Se in the flesh was significantly affected by Cu and Zn (*p* < 0.05). All dietary indicators were approximately 4.33 times higher for Se-rich navel oranges than for ordinary navel oranges. Se deficiency in the Chinese population remains a primary concern.

## Data availability statement

The original contributions presented in this study are included in the article/Supplementary material, further inquiries can be directed to the corresponding authors.

## Author contributions

FY contributed significantly to analysis and manuscript preparation, performed the data analyses, and wrote the manuscript. ZY and MY contributed to the conception of the study. LW, RC, CD, and SS helped to perform the analysis with constructive discussions. All authors contributed to the article and approved the submitted version.

## Footnotes

1
http://www.r-project.org/
