# Supplementary material for: Enrichment characteristics and dietary evaluation of selenium in navel orange fruit from the largest navel orange-producing area in China (southern Jiangxi)
Source: Front Plant Sci. 2022 Aug 8;13:881098. doi: 10.3389/fpls.2022.881098 (PMC9393740; doi:10.3389/fpls.2022.881098)
Supplement: Supplementary file 1 [file Data_Sheet_1.doc]

**Supplemental Table 1** Land area statistics of total selenium content in arable soils of southern Jiangxi Province, China.

| Regions | Arable land area (km^2^) | | | | |
| --- | --- | --- | --- | --- | --- |
|  | Total selenium content in soil (mg∙kg^-1^) | | | | |
|  | > 0.4 | 0.3-0.4 | 0.175-0.3 | 0.125-0.175 | < 0.125 |
| Zhanggong District | 8.34 | 47.18 | 105.95 | 1.1 | 0.57 |
| Nankang District | 166.71 | 191.72 | 518.69 | 133.05 | 38.92 |
| Ganxian District | 412.62 | 526.17 | 472.90 | 46.24 | 7.32 |
| Xinfeng County | 504.26 | 276.69 | 457.17 | 112.87 | 19.24 |
| Dayu County | 103.56 | 69.44 | 150.85 | 5.42 | 0.48 |
| Chongyi County | 426.02 | 91.29 | 24.61 | 0.12 | 0.02 |
| Shangyu County | 203.68 | 188.05 | 272.34 | 35.18 | 3.72 |
| Anyuan County | 136.61 | 174.07 | 265.98 | 59.31 | 13.90 |
| Longnan City | 190.87 | 149.58 | 189.44 | 18.56 | 0.90 |
| Dingnan County | 99.81 | 123.46 | 85.46 | 17.64 | 5.94 |
| Quanan County | 274.57 | 95.69 | 73.38 | 4.53 | 0.83 |
| Xingguo County | 291.43 | 320.29 | 683.00 | 212.45 | 25.10 |
| Ningdu County | 113.68 | 194.63 | 1198.79 | 549.24 | 88.35 |
| Yudu County | 618.69 | 393.84 | 740.92 | 171.33 | 22.65 |
| Ruijin City | 125.11 | 139.97 | 322.72 | 110.74 | 20.37 |
| Huichang County | 140.41 | 164.58 | 378.99 | 81.22 | 5.00 |
| Xunwu County | 296.92 | 184.03 | 213.60 | 22.90 | 2.42 |
| Shicheng County | 1.23 | 5.57 | 77.35 | 187.86 | 129.37 |
| Southern Jiangxi Region | 4114.51 | 3336.25 | 6232.14 | 1769.76 | 385.09 |

Notes: Data from 1:50,000 soil quality geochemical survey soil data in Southern Jiangxi Province, China, China Geological Survey (2019).

**Supplemental Table 2** Location of samples collected in southern Jiangxi Province, China, and content of selenium (Se), boron (B), manganese (Mn), iron (Fe), copper (Cu), zinc (Zn), molybdenum (Mo), magnesium (Mg), phosphorus (P), potassium (K) and calcium (Ca) in the navel orange flesh (n = 492).

| **Sample number** | **Regions** | **Longitude**  **(E)** | **Latitude**  **(N)** | **Se**  **(µg∙kg^-1^)** | **B (mg∙kg^-1^)** | **Mn**  **(mg∙kg^-1^)** | **Fe**  **(mg∙kg^-1^)** | **Cu**  **(mg∙kg^-1^)** | **Zn**  **(mg∙kg^-1^)** | **Mo**  **(mg∙kg^-1^)** | **Mg**  **(mg∙kg^-1^)** | **P**  **(mg∙kg^-1^)** | **K (mg∙kg^-1^)** | **Ca**  **(mg∙kg^-1^)** |
| --- | --- | --- | --- | --- | --- | --- | --- | --- | --- | --- | --- | --- | --- | --- |
| 1 | Zhanggong District | 114.97623 | 25.80168 | 3.3048 | 14.0340 | 5.4718 | 9.4578 | 3.2806 | 3.4523 | 0.0269 | 76.6 | 111.1 | 1249.1 | 187.5 |
| 2 |  | 114.89650 | 25.93380 | 10.3466 | 14.3817 | 8.4934 | 10.2355 | 2.3068 | 3.8211 | 0.0177 | 66.1 | 101.7 | 1069.5 | 199.2 |
| 3 |  | 114.91975 | 25.92952 | 3.9871 | 18.2748 | 3.2037 | 14.2494 | 3.3248 | 4.3287 | 0.0327 | 85.6 | 152.7 | 1358.7 | 257.5 |
| 4 |  | 114.94885 | 25.91469 | 1.4890 | 16.2475 | 7.1984 | 15.4927 | 2.7388 | 3.4874 | 0.0195 | 87.4 | 107.5 | 1104.1 | 203.3 |
| 5 |  | 114.93674 | 25.87123 | 2.0529 | 14.1497 | 8.6666 | 16.1214 | 3.0984 | 3.8037 | 0.0260 | 86.4 | 159.1 | 1182.4 | 346.4 |
| 6 |  | 114.90333 | 25.93952 | 2.8770 | 20.7806 | 3.7694 | 17.3809 | 2.8562 | 4.8530 | 0.0275 | 84.7 | 113.8 | 1331.8 | 262.5 |
| 7 |  | 114.70989 | 25.70368 | 3.8447 | 17.6246 | 5.1173 | 16.6623 | 3.6142 | 3.8735 | 0.0199 | 113.7 | 137.5 | 1295.1 | 275.8 |
| 8 |  | 114.84542 | 25.74700 | 2.9986 | 12.9097 | 3.8853 | 11.1605 | 3.3704 | 3.8616 | 0.0417 | 107.2 | 131.6 | 1276.2 | 244.9 |
| 9 |  | 114.91051 | 25.72786 | 6.0804 | 18.5872 | 8.4783 | 11.7784 | 3.0751 | 4.9487 | 0.0156 | 87.6 | 141.6 | 916.6 | 209.4 |
| 10 |  | 114.77695 | 25.78800 | 6.6937 | 14.5163 | 1.6760 | 10.6189 | 1.9984 | 2.1699 | 0.0168 | 86.6 | 123.5 | 1108.9 | 250.4 |
| 11 |  | 114.57092 | 26.18851 | 2.6591 | 11.3418 | 1.7697 | 9.4475 | 2.3470 | 2.3145 | 0.0221 | 89.3 | 101.5 | 988.1 | 186.6 |
| 12 | Nankang District | 114.70038 | 25.69853 | 2.3617 | 22.7648 | 4.4614 | 14.4147 | 2.8469 | 3.8249 | 0.0212 | 88.7 | 139.5 | 1218.9 | 279.7 |
| 13 |  | 114.67901 | 25.72909 | 5.1380 | 20.5769 | 3.1315 | 18.7880 | 2.6721 | 3.7173 | 0.0365 | 78.4 | 124.1 | 1101 | 231.9 |
| 14 |  | 114.71573 | 25.78818 | 3.0282 | 13.9099 | 2.6459 | 11.9766 | 2.6643 | 3.1786 | 0.0250 | 89.4 | 134.7 | 1303.4 | 545.1 |
| 15 |  | 114.64458 | 25.74923 | 7.3397 | 14.4246 | 3.7587 | 11.7021 | 3.2708 | 3.4791 | 0.0233 | 85.8 | 146.7 | 1384.5 | 306.5 |
| 16 |  | 114.69546 | 25.69811 | 4.7951 | 13.3162 | 1.9414 | 10.4118 | 2.7302 | 2.9321 | 0.0114 | 77.1 | 133.3 | 1142.6 | 184.6 |
| 17 |  | 114.70799 | 26.06739 | 5.9978 | 12.2948 | 16.4321 | 12.9188 | 3.5496 | 3.9928 | 0.0217 | 101.9 | 134.1 | 1228.7 | 315.9 |
| 18 |  | 114.70989 | 25.70368 | 4.2016 | 17.8072 | 3.9223 | 11.6359 | 2.2942 | 3.8899 | 0.0104 | 96.7 | 97.4 | 1044.8 | 238.1 |
| 19 |  | 114.66468 | 25.78882 | 2.0508 | 19.0933 | 6.3040 | 11.6147 | 2.3244 | 4.3339 | 0.0332 | 114.5 | 108.4 | 1243.6 | 325.5 |
| 20 |  | 114.76886 | 25.82135 | 2.6641 | 11.9521 | 2.8611 | 11.9433 | 2.4944 | 2.5633 | 0.0224 | 112.8 | 139.2 | 1180.3 | 250.3 |
| 21 |  | 114.77045 | 25.81872 | 4.4880 | 15.4593 | 2.9977 | 36.9408 | 2.1171 | 3.3931 | 0.0127 | 81.2 | 128 | 1223.2 | 142.3 |
| 22 |  | 114.90300 | 25.72139 | 3.7772 | 15.4707 | 1.5360 | 8.5931 | 2.3804 | 2.5594 | 0.0517 | 101.4 | 147.5 | 1092.6 | 249.4 |
| 23 |  | 114.86150 | 25.70398 | 2.2637 | 15.2917 | 2.1839 | 16.2123 | 2.0817 | 3.1822 | 0.0288 | 73.8 | 126.4 | 1283.9 | 189 |
| 24 | Ganxian District | 115.15212 | 26.07371 | 5.1522 | 15.0036 | 2.8583 | 12.1663 | 3.9462 | 4.4588 | 0.0246 | 84.6 | 114.9 | 1272.7 | 190.4 |
| 25 |  | 115.15815 | 26.05175 | 3.4927 | 13.5463 | 2.9586 | 12.8990 | 3.3150 | 3.5433 | 0.0358 | 84.9 | 143.2 | 1173 | 239.7 |
| 26 |  | 115.15815 | 26.05175 | 12.4132 | 14.8373 | 3.1111 | 10.7941 | 3.4807 | 4.8055 | 0.0217 | 77.7 | 107.5 | 929.8 | 189.2 |
| 27 |  | 115.16812 | 26.01613 | 5.9404 | 12.6248 | 3.5360 | 8.7271 | 1.4866 | 3.1405 | 0.0119 | 74.2 | 118.5 | 991.8 | 278.8 |
| 28 |  | 115.16670 | 26.01721 | 4.2330 | 13.4734 | 2.6376 | 13.5355 | 2.2122 | 3.6840 | 0.0405 | 89.2 | 123.6 | 1038.9 | 255.1 |
| 29 |  | 115.01609 | 25.62507 | 3.8586 | 7.7336 | 3.8988 | 10.0748 | 2.5220 | 2.2624 | 0.0106 | 57.1 | 84.3 | 791 | 252 |
| 30 |  | 114.93915 | 25.61989 | 15.2730 | 15.6655 | 4.0160 | 11.5617 | 3.3317 | 4.7084 | 0.0163 | 85.9 | 111.9 | 1191.8 | 279.9 |
| 31 |  | 115.00078 | 25.59314 | 9.0235 | 16.3289 | 12.2717 | 8.3236 | 2.3514 | 2.7761 | 0.0197 | 70.6 | 94.3 | 943.7 | 250.5 |
| 32 |  | 115.01017 | 25.51626 | 6.0909 | 29.7367 | 4.2125 | 17.4509 | 3.1614 | 5.0225 | 0.0265 | 98.3 | 139 | 1459.6 | 294.3 |
| 33 |  | 115.06115 | 25.55108 | 0.8744 | 16.3702 | 3.0056 | 12.9293 | 2.1009 | 3.0665 | 0.0179 | 97 | 137.6 | 1323.1 | 205.7 |
| 34 |  | 115.10953 | 25.46789 | 1.9011 | 20.2098 | 2.3045 | 16.2443 | 1.5690 | 3.5571 | 0.0497 | 88.9 | 122.6 | 1163.4 | 199.3 |
| 35 |  | 115.09608 | 25.46022 | 1.8643 | 16.0203 | 7.1399 | 12.3612 | 1.0976 | 3.4723 | 0.0187 | 79.6 | 101.6 | 1041.7 | 189.3 |
| 36 |  | 115.12665 | 25.48057 | 3.1164 | 16.5335 | 10.9726 | 14.3355 | 2.7999 | 3.3237 | 0.2252 | 76.2 | 113.9 | 1048.9 | 251.8 |
| 37 |  | 114.93915 | 25.61989 | 3.1209 | 17.8671 | 3.8814 | 16.5591 | 2.8328 | 3.4629 | 0.0467 | 84.5 | 143.9 | 1148.6 | 259.6 |
| 38 |  | 114.92459 | 25.61324 | 2.2622 | 20.0295 | 2.2611 | 13.8239 | 2.4157 | 3.6289 | 0.0330 | 99.2 | 147.7 | 1338.1 | 177.9 |
| 39 |  | 114.91665 | 25.62374 | 2.3841 | 16.9742 | 6.0736 | 12.2076 | 2.9474 | 5.3327 | 0.0186 | 118.6 | 150.6 | 1406.3 | 269.3 |
| 40 |  | 114.92424 | 25.64598 | 2.6492 | 20.7196 | 7.2319 | 12.5556 | 1.9850 | 3.4642 | 0.0188 | 73.9 | 99.5 | 1016.4 | 255.5 |
| 41 |  | 114.87282 | 25.63010 | 2.4355 | 17.7375 | 6.4850 | 13.7088 | 2.3215 | 3.0301 | 0.0239 | 97 | 144.7 | 1323.7 | 188.1 |
| 42 |  | 115.20462 | 26.08964 | 2.9112 | 2.0814 | 0.2619 | 7.4546 | 0.6819 | 0.2811 | 0.0072 | 8.9 | 3.8 | 84.6 | 48 |
| 43 |  | 115.24255 | 26.09483 | 2.3137 | 19.3513 | 3.9418 | 11.3875 | 2.8210 | 3.5655 | 0.0436 | 118 | 162.4 | 1571.5 | 260.9 |
| 44 |  | 115.24929 | 26.05498 | 1.3609 | 16.0408 | 2.3431 | 11.5410 | 3.0087 | 4.2845 | 0.0517 | 108.3 | 148 | 1358.6 | 278.4 |
| 45 |  | 115.25467 | 26.07400 | 2.2669 | 10.8642 | 3.7851 | 10.6488 | 2.1778 | 3.4001 | 0.0212 | 83 | 116.4 | 1135.6 | 183.6 |
| 46 |  | 115.18446 | 26.00160 | 5.6336 | 21.3763 | 2.1207 | 12.0121 | 2.4755 | 3.5170 | 0.0481 | 80.7 | 155.3 | 1062.5 | 270.6 |
| 47 |  | 115.25598 | 26.01350 | 4.0032 | 19.3220 | 3.3377 | 12.4817 | 2.8851 | 5.3586 | 0.0222 | 99.4 | 152.2 | 1358 | 200.5 |
| 48 |  | 115.14245 | 26.02976 | 1.9791 | 22.4114 | 5.4187 | 14.0896 | 1.2113 | 5.7139 | 0.0188 | 100.8 | 183.2 | 1489.2 | 197.1 |
| 49 |  | 115.31062 | 25.99861 | 1.9085 | 20.1103 | 3.8178 | 13.4410 | 2.6616 | 4.8763 | 0.0276 | 101.9 | 116.1 | 1149.7 | 228.3 |
| 50 | Xinfeng County  Xinfeng County | 114.79562 | 25.40781 | 6.4931 | 12.1063 | 3.3822 | 13.0507 | 3.0780 | 3.6149 | 0.0121 | 105 | 117.6 | 1046.2 | 199.6 |
| 51 |  | 114.78878 | 25.40336 | 3.3517 | 15.5018 | 7.0730 | 20.1208 | 3.3287 | 3.9930 | 0.0291 | 104.2 | 126.2 | 1111 | 248.7 |
| 52 |  | 115.67132 | 25.99760 | 7.3328 | 17.4942 | 3.2361 | 11.2793 | 2.0000 | 3.3938 | 0.0099 | 75.4 | 101.6 | 1077.9 | 226 |
| 53 |  | 114.80919 | 25.39581 | 4.6729 | 17.0674 | 3.8639 | 13.2845 | 2.4508 | 3.6256 | 0.0300 | 77.5 | 126.7 | 1142 | 252.6 |
| 54 |  | 114.80679 | 25.39399 | 2.1986 | 15.8406 | 3.0303 | 28.7474 | 3.7773 | 5.5373 | 0.0400 | 72.6 | 138.2 | 1069.9 | 351.2 |
| 55 |  | 114.74608 | 25.39197 | 2.4966 | 13.9794 | 3.8484 | 11.7171 | 2.6779 | 4.1589 | 0.0161 | 72.3 | 124.3 | 1057.6 | 337.7 |
| 56 |  | 114.82316 | 25.42666 | 6.5425 | 12.6546 | 3.2802 | 11.3511 | 2.9183 | 3.5714 | 0.0267 | 109.9 | 125.5 | 1143.1 | 167.8 |
| 57 |  | 114.83253 | 25.41941 | 2.4598 | 15.6462 | 6.6922 | 12.2032 | 3.3482 | 3.8604 | 0.0245 | 96.3 | 132.2 | 1133.7 | 219.5 |
| 58 |  | 115.22066 | 25.45188 | 2.7656 | 12.9684 | 3.6177 | 11.4118 | 2.6366 | 3.3015 | 0.0192 | 77.2 | 109.8 | 1234.8 | 153.8 |
| 59 |  | 114.82702 | 25.33539 | 2.8523 | 14.7558 | 3.9921 | 10.1668 | 2.2241 | 3.2595 | 0.0392 | 82.1 | 118.8 | 992.6 | 256.9 |
| 60 |  | 114.98496 | 25.08887 | 3.5626 | 17.3099 | 4.3908 | 10.1980 | 2.6023 | 3.9852 | 0.0228 | 77.3 | 101.4 | 1119.6 | 211.4 |
| 61 |  | 114.98500 | 25.08634 | 3.0324 | 14.0974 | 15.4190 | 9.4809 | 2.6878 | 2.8895 | 0.0330 | 85.4 | 132.3 | 1095.7 | 207 |
| 62 |  | 114.91977 | 25.10871 | 5.2064 | 19.1672 | 7.2484 | 15.2813 | 3.2669 | 4.8002 | 0.0208 | 85.1 | 128 | 1247.6 | 237.8 |
| 63 |  | 114.98252 | 25.08994 | 6.5285 | 21.6785 | 14.9103 | 11.2591 | 2.7346 | 4.7180 | 0.0139 | 85.5 | 117.5 | 1034.1 | 236.9 |
| 64 |  | 114.98475 | 25.08689 | 4.4282 | 20.7975 | 5.9853 | 12.6319 | 3.0966 | 5.3922 | 0.0258 | 86.3 | 120 | 979.8 | 238.2 |
| 65 |  | 114.97863 | 25.04549 | 4.0649 | 21.7540 | 3.5746 | 11.8521 | 2.0975 | 3.9265 | 0.0177 | 76.9 | 102.8 | 1065.5 | 278.3 |
| 66 |  | 114.93260 | 25.12503 | 7.9463 | 17.5697 | 2.0275 | 11.5648 | 3.7717 | 4.3614 | 0.0251 | 84.4 | 126.3 | 1085.9 | 184.2 |
| 67 |  | 114.97743 | 25.05579 | 5.3213 | 17.4649 | 6.8400 | 14.3319 | 2.6405 | 4.7247 | 0.0209 | 69.4 | 105.2 | 1067 | 250.4 |
| 68 |  | 114.96372 | 25.05987 | 2.8320 | 18.5726 | 7.9808 | 11.5557 | 2.4636 | 4.4167 | 0.0340 | 80.2 | 120.7 | 921.7 | 256.5 |
| 69 |  | 115.10101 | 25.18406 | 4.8116 | 12.9122 | 17.8879 | 9.8900 | 2.1938 | 2.9454 | 0.0230 | 87.4 | 103.7 | 867.2 | 264.5 |
| 70 |  | 115.11641 | 25.23374 | 5.1491 | 16.7688 | 3.9184 | 13.8549 | 3.0962 | 4.2594 | 0.0228 | 82.7 | 124.5 | 1134.9 | 229.3 |
| 71 |  | 115.21680 | 25.35919 | 2.4469 | 21.0273 | 5.6547 | 13.5472 | 2.0516 | 3.8367 | 0.0097 | 76.9 | 122.9 | 1129.3 | 248.5 |
| 72 |  | 114.92518 | 25.17531 | 6.6408 | 17.7890 | 2.2533 | 11.2205 | 2.1218 | 3.8139 | 0.0145 | 86.6 | 117.6 | 1017.8 | 218.9 |
| 73 |  | 114.93709 | 25.18021 | 39.8847 | 17.5434 | 4.9891 | 15.4048 | 2.0521 | 3.4007 | 0.0384 | 85 | 132 | 1166.3 | 183.6 |
| 74 |  | 114.92547 | 25.18908 | 6.3274 | 18.8988 | 5.8251 | 13.5445 | 1.7606 | 5.0746 | 0.0179 | 79.3 | 111.5 | 880.9 | 259.3 |
| 75 |  | 114.92776 | 25.18161 | 2.8102 | 20.3626 | 6.7120 | 13.3981 | 2.1172 | 4.1362 | 0.0062 | 84.8 | 134 | 1065.8 | 255.8 |
| 76 |  | 114.93667 | 25.18387 | 2.2947 | 20.2823 | 8.9580 | 10.4081 | 2.8413 | 3.0662 | 0.0061 | 94.8 | 77.6 | 1027.9 | 191.5 |
| 77 |  | 114.94074 | 25.18670 | 2.1759 | 20.4652 | 4.9392 | 12.1949 | 2.1899 | 3.5309 | 0.0129 | 85.4 | 109.3 | 1069.5 | 205.1 |
| 78 |  | 114.94095 | 25.18911 | 6.7876 | 19.6667 | 2.0534 | 14.7910 | 2.5083 | 3.1403 | 0.0203 | 81.5 | 130.1 | 931.6 | 277.9 |
| 79 |  | 114.94453 | 25.19030 | 2.9513 | 19.1206 | 5.7811 | 12.0353 | 1.9289 | 3.7114 | 0.0167 | 72 | 135.5 | 1098.4 | 202.8 |
| 80 |  | 114.95710 | 25.19208 | 4.5888 | 14.3795 | 12.1449 | 18.1191 | 3.5304 | 4.0770 | 0.0224 | 98.3 | 120.1 | 972 | 247.8 |
| 81 |  | 115.07259 | 25.29651 | 8.6449 | 20.8588 | 3.0048 | 10.9798 | 3.3554 | 4.6262 | 0.0148 | 76.8 | 107.4 | 1110.1 | 243 |
| 82 |  | 115.10543 | 25.33152 | 2.7112 | 31.7329 | 17.2151 | 14.1507 | 5.7976 | 7.6715 | 0.0223 | 171.3 | 216.9 | 1929.6 | 432 |
| 83 |  | 114.95143 | 25.38428 | 3.8684 | 22.3229 | 6.6099 | 13.3603 | 2.5731 | 4.8274 | 0.0269 | 99.4 | 139.9 | 1179.5 | 240.2 |
| 84 |  | 114.99151 | 25.38916 | 3.9314 | 21.9285 | 4.0950 | 16.8166 | 2.2481 | 5.3769 | 0.0307 | 90.2 | 119.4 | 1211.1 | 209.9 |
| 85 |  | 115.01291 | 25.39794 | 2.4654 | 20.8411 | 6.9967 | 14.4368 | 2.8122 | 6.3848 | 0.0210 | 100.8 | 110.6 | 1095.1 | 254.2 |
| 86 |  | 114.94540 | 25.34479 | 2.6559 | 17.4658 | 11.3521 | 16.4251 | 2.4527 | 4.6999 | 0.0314 | 108.7 | 147.3 | 1590 | 280.5 |
| 87 |  | 114.88471 | 25.42156 | 14.4157 | 19.3341 | 5.0900 | 16.6014 | 2.2382 | 5.7577 | 0.0412 | 85.6 | 104.5 | 954.7 | 250.9 |
| 88 |  | 115.00688 | 25.48276 | 4.4410 | 19.0461 | 4.9349 | 15.2066 | 3.2407 | 5.3754 | 0.0620 | 108.3 | 197.7 | 1430.4 | 405 |
| 89 |  | 114.87339 | 25.45694 | 3.0682 | 22.2824 | 5.4758 | 12.8978 | 2.7434 | 4.8577 | 0.0494 | 79.2 | 107.2 | 848.6 | 281.2 |
| 90 |  | 114.87025 | 25.49024 | 3.2011 | 24.0948 | 5.7543 | 14.2630 | 2.8021 | 5.5760 | 0.0191 | 99.7 | 159.8 | 1221.1 | 311 |
| 91 |  | 114.78653 | 25.43437 | 5.0025 | 18.0952 | 5.2914 | 12.8154 | 2.0330 | 4.2757 | 0.0471 | 111 | 153.4 | 1561.1 | 233.4 |
| 92 |  | 115.21680 | 25.35919 | 3.0051 | 21.0225 | 4.7840 | 21.9704 | 2.5541 | 4.4758 | 0.0145 | 85.3 | 149.6 | 961.4 | 253 |
| 93 |  | 115.22771 | 25.40003 | 6.2126 | 20.6929 | 2.7966 | 12.6661 | 2.6326 | 3.9155 | 0.0114 | 78.7 | 103.8 | 1074.4 | 230.4 |
| 94 |  | 115.21196 | 25.36406 | 4.1349 | 21.9728 | 3.5678 | 13.3271 | 2.0399 | 4.1596 | 0.0206 | 87.2 | 112.3 | 957.9 | 321.1 |
| 95 | Dayu County  Dayu County | 114.55869 | 25.48448 | 5.1552 | 14.8533 | 2.9117 | 10.5940 | 2.6994 | 3.6670 | 0.0391 | 67.6 | 106.7 | 807 | 235.6 |
| 96 |  | 114.56540 | 25.50865 | 3.9505 | 13.4021 | 6.7376 | 12.9574 | 1.9520 | 4.2848 | 0.0268 | 86.8 | 115.5 | 973.3 | 198.4 |
| 97 |  | 114.56788 | 25.50948 | 8.2815 | 13.2436 | 5.8782 | 13.2424 | 3.1792 | 5.1606 | 0.0241 | 92.3 | 115.3 | 1027.7 | 253.9 |
| 98 |  | 114.57035 | 25.50921 | 3.5831 | 9.6005 | 3.8120 | 10.8572 | 1.8768 | 5.0562 | 0.0087 | 78.9 | 116.3 | 939.4 | 198.4 |
| 99 |  | 114.56996 | 25.50889 | 3.9583 | 13.4687 | 4.3984 | 13.4593 | 3.0413 | 5.1172 | 0.0131 | 91.7 | 120.7 | 1110.2 | 214.7 |
| 100 |  | 114.55489 | 25.48542 | 3.2624 | 12.6156 | 4.4599 | 15.7386 | 2.6690 | 3.8555 | 0.0038 | 82 | 123 | 1020.1 | 209.1 |
| 101 |  | 114.60864 | 25.51969 | 2.4825 | 10.1298 | 2.5058 | 10.9312 | 4.0250 | 4.0478 | 0.0277 | 62.6 | 100.3 | 790.1 | 217.1 |
| 102 |  | 114.56206 | 25.48852 | 3.0308 | 16.9729 | 6.3762 | 10.2699 | 3.3082 | 6.1536 | 0.0308 | 74.2 | 129.8 | 835.4 | 289.4 |
| 103 |  | 114.51515 | 25.46409 | 2.9870 | 10.9924 | 2.9250 | 10.0256 | 1.9782 | 3.2917 | 0.0487 | 79.9 | 89 | 1008.6 | 245.8 |
| 104 |  | 114.54594 | 25.47809 | 2.9618 | 13.1150 | 2.8565 | 8.9072 | 3.2375 | 5.0962 | 0.0055 | 87.3 | 112.2 | 1087.8 | 260.4 |
| 105 |  | 114.51515 | 25.46409 | 5.7747 | 13.0818 | 2.5380 | 9.2470 | 2.5132 | 3.0927 | 0.0402 | 102.6 | 128.5 | 1090.3 | 192.8 |
| 106 |  | 114.55895 | 25.47740 | 4.2627 | 16.4938 | 3.0567 | 12.9149 | 4.1575 | 5.3972 | 0.0234 | 94.3 | 135.8 | 1124.1 | 327.1 |
| 107 |  | 114.57806 | 25.49423 | 2.9152 | 17.8441 | 9.2268 | 12.9669 | 4.1037 | 5.2325 | 0.0247 | 81.8 | 123 | 1200.4 | 314.2 |
| 108 |  | 114.56089 | 25.48667 | 3.6868 | 14.6058 | 7.0028 | 10.4290 | 3.0730 | 5.0723 | 0.0152 | 98.6 | 121.9 | 1039.2 | 292.4 |
| 109 |  | 114.57160 | 25.48752 | 8.3124 | 15.3933 | 2.9239 | 13.3954 | 4.6848 | 4.5384 | 0.0320 | 92.4 | 147.8 | 1121.9 | 251.5 |
| 110 |  | 114.54623 | 25.48288 | 3.9465 | 13.8233 | 2.9772 | 12.8184 | 4.4046 | 5.1509 | 0.0251 | 91.1 | 109 | 1177.4 | 265.3 |
| 111 |  | 114.55191 | 25.48523 | 4.5391 | 16.9144 | 3.0915 | 12.8974 | 2.2404 | 5.2600 | 0.0288 | 87.7 | 134.5 | 1042.7 | 318.6 |
| 112 |  | 114.57052 | 25.49358 | 5.9959 | 13.1790 | 3.6774 | 11.7210 | 3.2984 | 5.2282 | 0.0148 | 100.6 | 144.2 | 1076.9 | 226.4 |
| 113 |  | 114.57799 | 25.48210 | 10.9291 | 14.0864 | 3.3254 | 11.6093 | 1.9259 | 3.5945 | 0.0403 | 80.9 | 115.4 | 856.4 | 255.3 |
| 114 |  | 114.54646 | 25.51338 | 3.7951 | 17.2559 | 4.3764 | 14.2731 | 2.4006 | 5.2476 | 0.0167 | 95.5 | 116.7 | 1066.8 | 293.7 |
| 115 |  | 114.56503 | 25.52555 | 2.4611 | 17.8625 | 3.9867 | 13.6628 | 3.0718 | 4.9870 | 0.0140 | 99.8 | 112.2 | 1208.4 | 247.9 |
| 116 |  | 114.55626 | 25.51499 | 3.5447 | 15.3948 | 4.1869 | 13.1340 | 3.5320 | 4.4738 | 0.0196 | 103.6 | 171 | 1259.2 | 390.6 |
| 117 |  | 114.56431 | 25.51877 | 4.6690 | 14.7758 | 2.9299 | 13.0659 | 3.8603 | 4.9609 | 0.0201 | 110.6 | 181 | 1438.4 | 256.1 |
| 118 |  | 114.54319 | 25.52425 | 1.9260 | 14.2000 | 5.1040 | 12.4483 | 2.3864 | 3.9726 | 0.0698 | 101.8 | 170.3 | 1370.6 | 273.8 |
| 119 |  | 114.55138 | 25.51603 | 4.9189 | 18.8976 | 5.6684 | 13.5834 | 2.1897 | 4.9684 | 0.0396 | 124.6 | 152.8 | 1192 | 307.6 |
| 120 |  | 114.56589 | 25.52060 | 2.3081 | 13.4079 | 6.6922 | 14.8360 | 2.4467 | 3.6407 | 0.1319 | 74.2 | 85.4 | 946.9 | 260.4 |
| 121 |  | 114.52449 | 25.51034 | 5.3531 | 16.5010 | 7.0694 | 11.9274 | 1.6962 | 3.9210 | 0.0255 | 99.8 | 129.3 | 1091 | 188.1 |
| 122 |  | 114.44886 | 25.46530 | 3.9483 | 17.4617 | 6.2778 | 10.3537 | 3.3640 | 3.5143 | 0.0185 | 78.3 | 107.4 | 992.5 | 311.6 |
| 123 |  | 114.45264 | 25.46593 | 1.3939 | 13.3311 | 3.2024 | 11.0895 | 2.1252 | 3.4972 | 0.0089 | 81 | 117.2 | 1045.9 | 200.6 |
| 124 |  | 114.44553 | 25.44073 | 3.9020 | 13.0232 | 2.7014 | 9.8531 | 3.9360 | 4.3580 | 0.0266 | 76.9 | 113.2 | 890.3 | 217.8 |
| 125 |  | 114.43047 | 25.43574 | 3.9117 | 16.2273 | 5.1194 | 9.8353 | 2.1016 | 3.5524 | 0.0229 | 85.7 | 107.4 | 1030 | 196.5 |
| 126 |  | 114.53041 | 25.48965 | 4.5723 | 15.3042 | 3.4901 | 12.4570 | 3.8942 | 5.0235 | 0.0200 | 80.8 | 117.5 | 1050.5 | 203.9 |
| 127 |  | 114.47861 | 25.44731 | 3.9107 | 18.5773 | 3.2533 | 11.0857 | 4.0632 | 5.4994 | 0.0841 | 76.4 | 115.8 | 894.6 | 165.6 |
| 128 |  | 114.54918 | 25.48888 | 2.5481 | 23.8648 | 2.9886 | 17.2436 | 4.2936 | 4.7650 | 0.0308 | 114.2 | 146.2 | 1306.6 | 190.6 |
| 129 |  | 114.56540 | 25.50865 | 1.5166 | 21.8521 | 4.0693 | 19.2851 | 5.5950 | 5.3162 | 0.0219 | 102.1 | 153.9 | 1310.7 | 235.7 |
| 130 |  | 114.55008 | 25.51264 | 1.2605 | 22.0667 | 5.4550 | 14.6166 | 3.2130 | 3.9081 | 0.0118 | 88.6 | 117.6 | 1200.4 | 189.9 |
| 131 |  | 114.53399 | 25.52399 | 2.2261 | 18.3588 | 4.1644 | 17.3765 | 3.4236 | 4.4931 | 0.0119 | 164.5 | 137.5 | 1079.5 | 331.4 |
| 132 |  | 114.59588 | 40.40885 | 3.0588 | 20.8920 | 2.1614 | 17.2239 | 3.4670 | 4.1809 | 0.0365 | 124.4 | 125.4 | 1151.3 | 365 |
| 133 |  | 114.49690 | 25.52060 | 1.5667 | 23.3260 | 3.5325 | 18.3071 | 4.1585 | 6.4434 | 0.0289 | 141.1 | 156.2 | 1488.1 | 219.5 |
| 134 |  | 114.48843 | 25.57671 | 5.9343 | 22.3908 | 4.5239 | 16.2710 | 3.0056 | 4.5194 | 0.0320 | 121.4 | 117.1 | 1052.3 | 298.1 |
| 135 |  | 114.57862 | 25.49290 | 3.2529 | 19.9904 | 3.3039 | 14.2857 | 1.7988 | 4.0024 | 0.0117 | 84.7 | 137.3 | 1327.5 | 123.7 |
| 136 |  | 114.52368 | 25.52399 | 1.1734 | 18.6201 | 5.0568 | 11.9457 | 3.5378 | 3.8980 | 0.0141 | 80.8 | 90.3 | 1001.7 | 177.2 |
| 137 |  | 114.55371 | 25.52907 | 2.8976 | 20.3580 | 3.9734 | 14.2273 | 2.0864 | 4.0627 | 0.0256 | 108.1 | 156.6 | 1377.2 | 340.4 |
| 138 |  | 114.49639 | 25.50846 | 3.0776 | 16.6009 | 3.8937 | 13.4424 | 2.2428 | 4.5712 | 0.0310 | 104.2 | 144.9 | 1099.2 | 287.2 |
| 139 |  | 114.45709 | ,25.48582 | 3.6697 | 17.2781 | 6.6250 | 20.4214 | 2.5255 | 6.0781 | 0.0265 | 99.8 | 149.1 | 1269.4 | 262.8 |
| 140 |  | 114.46190 | 25.48301 | 2.2593 | 19.1411 | 4.1417 | 14.2865 | 1.9278 | 3.5454 | 0.0413 | 113.9 | 131.7 | 1427.7 | 226.9 |
| 141 |  | 114.36943 | 25.46490 | 4.0754 | 19.4280 | 4.4095 | 13.6305 | 2.2319 | 4.2271 | 0.0886 | 101.2 | 149.2 | 1529.8 | 297.2 |
| 142 |  | 114.25227 | 25.33605 | 1.7184 | 12.1701 | 3.8920 | 10.0819 | 2.2059 | 3.2540 | 0.0162 | 102.9 | 149.6 | 1359.3 | 241.2 |
| 143 | Shangyu County | 114.44653 | 25.87790 | 35.5337 | 16.0926 | 7.7002 | 13.0404 | 1.6403 | 3.3766 | 0.0143 | 102.3 | 131.7 | 1140.7 | 277.9 |
| 144 |  | 114.49951 | 25.97190 | 2.1187 | 10.9050 | 6.3017 | 13.9555 | 2.5062 | 3.2085 | 0.0081 | 96.6 | 129.8 | 1254.1 | 185.2 |
| 145 |  | 114.56200 | 25.84464 | 3.2332 | 19.3350 | 5.0797 | 12.0089 | 1.8036 | 3.9937 | 0.0168 | 83.8 | 191 | 1197.9 | 178.4 |
| 146 |  | 114.46335 | 25.86198 | 2.5108 | 16.3731 | 5.2162 | 12.2138 | 1.4162 | 4.7489 | 0.0202 | 77.4 | 127.9 | 995.9 | 232.8 |
| 147 |  | 114.55436 | 25.83105 | 3.1956 | 19.3237 | 19.4238 | 24.5339 | 2.5989 | 4.9244 | 0.0255 | 103.6 | 145.2 | 1073.6 | 322.5 |
| 148 |  | 114.59116 | 25.80607 | 5.6595 | 17.2910 | 4.2993 | 6.2013 | 2.3001 | 3.9823 | 0.0472 | 82.3 | 114.6 | 1068.3 | 283 |
| 149 |  | 114.42048 | 25.81100 | 3.8750 | 15.1437 | 3.6940 | 8.2234 | 2.2539 | 3.0962 | 0.0312 | 72.3 | 100.3 | 1073.1 | 153.9 |
| 150 |  | 114.43600 | 25.80996 | 3.6973 | 14.3972 | 7.4779 | 10.7861 | 2.4691 | 4.3962 | 0.0556 | 89.2 | 292.8 | 1082.1 | 390.8 |
| 151 |  | 114.60163 | 25.95525 | 2.4475 | 21.3068 | 5.0680 | 12.8705 | 3.2360 | 4.1385 | 0.0143 | 91 | 124 | 1092.2 | 248.9 |
| 152 |  | 114.59301 | 25.93705 | 2.6250 | 17.6167 | 32.6683 | 9.8624 | 1.9922 | 3.4911 | 0.0157 | 98.4 | 105.7 | 1136.3 | 239.1 |
| 153 |  | 114.28447 | 25.92533 | 2.4942 | 15.8662 | 7.7625 | 11.7482 | 2.3647 | 3.5228 | 0.0133 | 88.9 | 131.5 | 1404.9 | 133 |
| 154 |  | 114.60317 | 25.79532 | 2.5869 | 18.8546 | 3.3538 | 10.9406 | 1.9868 | 3.3901 | 0.0140 | 83.4 | 131.8 | 1255 | 225 |
| 155 |  | 114.44546 | 25.99018 | 3.3501 | 21.1043 | 5.1982 | 12.5057 | 2.4484 | 3.8398 | 0.0130 | 104.8 | 102.1 | 1342.7 | 258.4 |
| 156 |  | 114.37302 | 26.02063 | 2.4572 | 17.5938 | 10.1524 | 20.2607 | 2.7306 | 3.6689 | 0.0239 | 82.7 | 93.4 | 901.6 | 218.5 |
| 157 |  | 114.33770 | 25.96080 | 3.1121 | 13.4255 | 3.0627 | 9.1978 | 2.3115 | 3.1090 | 0.0113 | 70.9 | 109.5 | 934.8 | 110.3 |
| 158 |  | 114.24670 | 25.91084 | 5.2728 | 14.2534 | 4.5372 | 19.9816 | 2.2081 | 4.1457 | 0.0173 | 95.5 | 112.6 | 1173.5 | 246.6 |
| 159 | Chongyi County  Chongyi County | 113.99107 | 25.76105 | 4.7608 | 13.2895 | 6.0976 | 11.1308 | 2.9908 | 3.0403 | 0.0094 | 94 | 112.1 | 1100.2 | 235.5 |
| 160 |  | 114.01842 | 25.73220 | 5.5010 | 16.0895 | 3.9572 | 12.1205 | 4.2467 | 3.9265 | 0.0214 | 104.2 | 120.4 | 1178 | 438.2 |
| 161 |  | 114.09488 | 25.65602 | 15.7205 | 12.4728 | 3.2278 | 12.8423 | 3.1199 | 2.9812 | 0.0213 | 97 | 136.1 | 1200.9 | 276.2 |
| 162 |  | 114.07930 | 25.66459 | 34.6944 | 14.0514 | 5.9938 | 10.3480 | 2.8839 | 3.5209 | 0.0886 | 86.8 | 131.2 | 984.8 | 220 |
| 163 |  | 114.09948 | 25.66302 | 3.6748 | 11.0940 | 4.1585 | 9.5768 | 2.1926 | 2.8407 | 0.0102 | 89.5 | 115.2 | 1011 | 211.7 |
| 164 |  | 114.10636 | 25.65740 | 1.7018 | 14.1129 | 5.1322 | 12.5164 | 2.4513 | 2.4929 | 0.0061 | 100.2 | 122.8 | 1118.5 | 303.5 |
| 165 |  | 114.09959 | 25.66257 | 2.2798 | 14.1455 | 5.7476 | 10.8668 | 3.2438 | 3.4780 | 0.0208 | 107.2 | 128.6 | 869.7 | 320.9 |
| 166 |  | 114.10119 | 25.66034 | 5.8153 | 11.9178 | 5.3445 | 11.8969 | 2.6563 | 3.9099 | 0.0113 | 88 | 117.4 | 956.8 | 241.3 |
| 167 |  | 114.11527 | 25.67624 | 2.0655 | 16.2569 | 8.5600 | 10.7355 | 2.8173 | 2.9953 | 0.0609 | 105.4 | 129.5 | 1063.1 | 302.4 |
| 168 |  | 114.10464 | 25.66165 | 7.0500 | 14.6898 | 3.4297 | 10.8622 | 2.1051 | 2.8567 | 0.0198 | 90.2 | 137.5 | 897.1 | 238.6 |
| 169 |  | 114.09461 | 25.66574 | 7.2078 | 17.8731 | 4.4532 | 11.9896 | 3.4782 | 4.5685 | 0.0297 | 106.4 | 168.9 | 1246.9 | 208.4 |
| 170 |  | 114.09716 | 25.67363 | 4.0782 | 13.9191 | 4.4685 | 11.1216 | 2.4863 | 3.1257 | 0.0195 | 100.3 | 123.9 | 1087.1 | 259.2 |
| 171 |  | 114.09817 | 25.66074 | 5.7942 | 14.4879 | 3.2620 | 9.3093 | 1.6575 | 2.9808 | 0.0240 | 78.4 | 110.8 | 924.9 | 229 |
| 172 |  | 114.09960 | 25.67611 | 2.1303 | 11.4454 | 3.8576 | 13.0567 | 2.5087 | 3.5293 | 0.0159 | 127.7 | 123.2 | 1136 | 237.5 |
| 173 |  | 114.13381 | 25.66452 | 1.7621 | 13.4592 | 4.3445 | 11.1652 | 3.2091 | 3.9656 | 0.0243 | 86.8 | 140.1 | 1039.2 | 240.9 |
| 174 |  | 115.00683 | 25.52153 | 2.1005 | 14.8885 | 7.5341 | 10.5414 | 2.6640 | 3.5268 | 0.0462 | 72.9 | 141.3 | 1061 | 241.2 |
| 175 |  | 115.03400 | 24.88150 | 3.1706 | 16.2998 | 5.9070 | 25.3298 | 3.0667 | 4.2481 | 0.0436 | 71.1 | 109.2 | 968.1 | 261.9 |
| 176 |  | 114.10041 | 25.66433 | 8.2956 | 16.0350 | 4.8241 | 10.0082 | 2.1747 | 3.0219 | 0.0635 | 74.1 | 115.4 | 1059.8 | 222.1 |
| 177 |  | 114.14874 | 25.80595 | 3.3813 | 15.3092 | 2.9446 | 11.4026 | 2.2702 | 3.1127 | 0.0120 | 100.3 | 134.9 | 1164.8 | 230.7 |
| 178 |  | 114.11759 | 25.83373 | 2.9852 | 12.0807 | 4.7300 | 10.3604 | 2.3177 | 2.6301 | 0.0252 | 72.6 | 102.3 | 771.4 | 235.5 |
| 179 |  | 114.13926 | 25.82066 | 4.4178 | 14.9203 | 4.5868 | 10.8376 | 2.6303 | 3.2377 | 0.0142 | 74.4 | 130.5 | 959.4 | 242 |
| 180 |  | 114.13720 | 25.83455 | 2.1357 | 10.6588 | 6.1914 | 8.5979 | 2.0129 | 2.6096 | 0.0034 | 71.9 | 114.9 | 1118.6 | 229.6 |
| 181 |  | 115.68069 | 26.11345 | 3.5183 | 13.4592 | 6.7599 | 10.2836 | 1.7764 | 2.8992 | 0.0116 | 70.1 | 95.6 | 896.8 | 162.2 |
| 182 |  | 114.12226 | 25.81191 | 9.0664 | 12.7270 | 4.0426 | 10.7819 | 3.3789 | 3.6035 | 0.0100 | 87.4 | 133.8 | 1299.6 | 200.5 |
| 183 |  | 114.34129 | 25.74233 | 1.8119 | 17.5050 | 8.2897 | 9.4094 | 2.5820 | 3.4973 | 0.0224 | 94.1 | 124.9 | 1382.1 | 315.5 |
| 184 |  | 114.21403 | 25.72369 | 2.8905 | 21.0343 | 3.6276 | 10.8292 | 2.1426 | 3.8104 | 0.0295 | 90.9 | 96.1 | 1292.4 | 272.3 |
| 185 |  | 115.25313 | 26.29089 | 4.2204 | 21.2355 | 7.0387 | 12.2067 | 2.7270 | 3.7694 | 0.0384 | 103.8 | 146.3 | 1226.2 | 232 |
| 186 |  | 114.44878 | 25.63545 | 2.0507 | 13.4291 | 3.9918 | 11.9937 | 1.3492 | 3.0798 | 0.0090 | 85.2 | 125.3 | 1419.5 | 156.4 |
| 187 |  | 114.76051 | 25.33444 | 2.2865 | 20.0927 | 6.4128 | 11.7515 | 2.0170 | 4.5945 | 0.0176 | 101.7 | 125.6 | 1146.1 | 241.7 |
| 188 |  | 114.54189 | 25.68992 | 1.7841 | 19.7775 | 10.1043 | 11.2731 | 2.2521 | 3.9852 | 0.0190 | 91.9 | 115.4 | 1291.8 | 244.1 |
| 189 | Anyuan County  Anyuan County  Anyuan County | 115.41571 | 25.35886 | 1.5297 | 13.0057 | 2.6368 | 8.6002 | 2.2935 | 3.1687 | 0.0077 | 74.9 | 83.1 | 931.5 | 155.8 |
| 190 |  | 115.41599 | 25.35642 | 1.8786 | 12.3086 | 14.3578 | 7.9122 | 2.6813 | 3.0647 | 0.0164 | 82.3 | 113.6 | 1075.9 | 147.7 |
| 191 |  | 115.42453 | 25.34071 | 4.6245 | 11.9271 | 6.1783 | 11.0943 | 1.8102 | 4.7930 | 0.0302 | 82.5 | 93.9 | 905.2 | 152.1 |
| 192 |  | 115.40696 | 24.93784 | 2.1274 | 9.8007 | 2.0662 | 8.0394 | 2.0213 | 4.0678 | 0.0243 | 68.7 | 107.1 | 911 | 134.1 |
| 193 |  | 115.41772 | 25.24744 | 1.2450 | 16.8510 | 8.9927 | 9.7157 | 1.7170 | 4.0093 | 0.0072 | 88.3 | 112.9 | 1061.1 | 157.3 |
| 194 |  | 115.26369 | 24.96743 | 1.2584 | 17.8895 | 7.1907 | 12.1407 | 1.7181 | 4.0606 | 0.0106 | 87.9 | 135 | 1059.9 | 157.1 |
| 195 |  | 115.32032 | 24.90323 | 1.2741 | 19.4924 | 7.0151 | 10.2005 | 2.0242 | 3.7176 | 0.0054 | 78.1 | 121.7 | 943.8 | 217.2 |
| 196 |  | 115.42343 | 25.33985 | 3.9691 | 15.3326 | 6.8915 | 8.8020 | 1.6187 | 3.8793 | 0.0124 | 85.3 | 116.5 | 1098.7 | 163.1 |
| 197 |  | 115.50750 | 25.45357 | 1.0625 | 18.1819 | 4.3199 | 11.5314 | 2.9369 | 4.8578 | 0.0105 | 112.1 | 131.2 | 1185.3 | 328.7 |
| 198 |  | 115.41948 | 25.33811 | 1.1165 | 14.3675 | 8.2074 | 19.7654 | 1.0022 | 3.0586 | 0.0049 | 83 | 114.8 | 976.7 | 231.7 |
| 199 |  | 115.41571 | 25.35886 | 3.4676 | 13.1763 | 5.2644 | 17.6184 | 2.0390 | 5.0682 | 0.0164 | 91.2 | 103.2 | 1041.8 | 198.7 |
| 200 |  | 115.41674 | 25.33431 | 1.6426 | 16.3358 | 6.6809 | 10.3866 | 1.5406 | 3.4743 | 0.0044 | 81.4 | 131.6 | 1041.3 | 161.6 |
| 201 |  | 115.41873 | 25.33576 | 1.2949 | 17.3923 | 6.8825 | 8.9297 | 1.8416 | 3.6059 | 0.0135 | 96 | 147.6 | 1035.7 | 122.4 |
| 202 |  | 115.42167 | 25.33567 | 1.8650 | 15.5423 | 8.5883 | 8.3003 | 2.0907 | 3.8041 | 0.0137 | 87.3 | 99 | 962 | 232.8 |
| 203 |  | 115.42030 | 25.33539 | 1.2358 | 15.2931 | 15.1916 | 27.0304 | 1.4642 | 3.1218 | 0.0154 | 80.7 | 78.5 | 1024.7 | 249.5 |
| 204 |  | 115.26053 | 25.06123 | 1.4753 | 17.7790 | 8.6274 | 11.1042 | 2.0288 | 4.4420 | 0.0123 | 105.3 | 119.8 | 1241.1 | 218.4 |
| 205 |  | 115.49044 | 25.10650 | 2.1189 | 14.2576 | 6.7032 | 8.6621 | 1.6421 | 3.2378 | 0.0073 | 119 | 144.6 | 1359.1 | 439.4 |
| 206 |  | 115.45025 | 25.41486 | 1.5705 | 20.0318 | 8.5895 | 10.3686 | 2.2271 | 4.4913 | 0.0111 | 92.7 | 136.6 | 999.6 | 185.6 |
| 207 |  | 115.42030 | 25.33539 | 1.1146 | 16.4105 | 9.5491 | 9.3436 | 1.6283 | 3.4993 | 0.0129 | 78.6 | 93.4 | 901.6 | 180.8 |
| 208 |  | 115.36442 | 25.36327 | 1.8829 | 19.1914 | 10.4212 | 11.0333 | 1.9990 | 4.5676 | 0.0081 | 111.1 | 155.7 | 1244.7 | 188 |
| 209 |  | 115.39697 | 25.37173 | 14.1663 | 16.4960 | 5.7625 | 13.2752 | 0.9924 | 3.7747 | 0.0083 | 84.6 | 108.3 | 1196.5 | 172.1 |
| 210 |  | 115.34688 | 24.98407 | 15.4315 | 13.5922 | 2.8637 | 9.3576 | 1.4554 | 3.4268 | 0.0020 | 71.4 | 112.5 | 1090.5 | 181.6 |
| 211 |  | 115.34069 | 24.97743 | 32.0569 | 9.1172 | 2.9846 | 9.0096 | 0.9273 | 2.2394 | 0.0097 | 84.1 | 100 | 1038.2 | 267.8 |
| 212 |  | 115.34039 | 24.97760 | 13.5937 | 11.7380 | 7.1098 | 9.5538 | 2.1273 | 3.3647 | 0.0072 | 73.6 | 107.6 | 836.6 | 187.8 |
| 213 |  | 115.37501 | 25.17666 | 2.4523 | 9.7411 | 5.5927 | 8.3037 | 2.1693 | 2.1213 | 0.0162 | 63.1 | 87.5 | 672.6 | 90.8 |
| 214 |  | 115.38492 | 25.15356 | 2.3138 | 17.7686 | 7.8701 | 11.1184 | 3.1456 | 3.2893 | 0.0194 | 101.1 | 133.2 | 1048.6 | 187.5 |
| 215 |  | 115.39339 | 25.16269 | 3.9755 | 15.3850 | 5.6948 | 11.1159 | 2.7023 | 3.5174 | 0.0058 | 86.4 | 116.5 | 1133.9 | 198 |
| 216 |  | 115.38396 | 25.15449 | 2.9836 | 12.4122 | 5.3839 | 10.1509 | 2.6184 | 2.6036 | 0.0217 | 73.9 | 89.4 | 752.8 | 139.4 |
| 217 |  | 115.37951 | 25.20449 | 2.9380 | 15.1175 | 6.5086 | 9.4047 | 2.5407 | 2.7874 | 0.0172 | 88.1 | 135.2 | 905.9 | 210.1 |
| 218 |  | 115.55868 | 25.30467 | 7.0531 | 12.5549 | 6.3810 | 8.6123 | 1.9575 | 2.5269 | 0.0114 | 79.6 | 98.9 | 836.9 | 214.6 |
| 219 |  | 115.40056 | 25.14274 | 3.3403 | 13.7124 | 8.7905 | 11.0411 | 2.7419 | 3.0974 | 0.0162 | 104.2 | 124.8 | 1110.3 | 233.9 |
| 220 |  | 115.37554 | 25.18882 | 2.7082 | 15.5276 | 6.7344 | 10.3557 | 2.9414 | 3.3627 | 0.0186 | 85.5 | 124.4 | 1031.2 | 176 |
| 221 |  | 115.38517 | 25.16152 | 8.6991 | 12.8440 | 7.7261 | 11.0740 | 2.9474 | 2.8715 | 0.0306 | 91.6 | 121.3 | 847.5 | 240.2 |
| 222 |  | 115.38706 | 25.17942 | 2.7246 | 19.4643 | 8.9847 | 13.1499 | 3.6257 | 4.4420 | 0.0277 | 102.6 | 150.4 | 1151.2 | 253.5 |
| 223 |  | 115.39245 | 25.16219 | 8.4074 | 14.0234 | 5.9948 | 12.0622 | 2.2023 | 2.5306 | 0.0256 | 78.3 | 103 | 886.2 | 142.9 |
| 224 |  | 115.44004 | 25.08505 | 3.2400 | 17.8808 | 8.3490 | 14.9425 | 2.8156 | 3.5788 | 0.0161 | 95.4 | 140.3 | 1036.1 | 233.2 |
| 225 |  | 115.38901 | 25.14993 | 3.5356 | 15.9922 | 7.4225 | 11.6566 | 2.7475 | 3.0433 | 0.0207 | 91.8 | 123.5 | 888.3 | 193.4 |
| 226 |  | 115.38236 | 25.15127 | 2.6300 | 12.5034 | 7.8371 | 17.1256 | 2.8763 | 2.6133 | 0.0364 | 94 | 104.4 | 975.9 | 206 |
| 227 |  | 115.37371 | 25.15888 | 1.9514 | 13.5417 | 5.6990 | 11.5184 | 3.0033 | 3.2648 | 0.0507 | 83.4 | 100.5 | 910.2 | 253.6 |
| 228 |  | 115.37172 | 25.15605 | 7.6372 | 15.0689 | 9.9420 | 16.3019 | 3.5856 | 5.4568 | 0.0414 | 107.5 | 138.5 | 980 | 344.3 |
| 229 |  | 115.37204 | 25.15313 | 2.9322 | 13.8500 | 5.0390 | 18.3671 | 2.7360 | 2.9704 | 0.0211 | 80.1 | 133.6 | 931.4 | 161.9 |
| 230 |  | 115.37393 | 25.14906 | 11.0399 | 12.4786 | 7.2581 | 11.2300 | 2.9498 | 2.7690 | 0.0256 | 76.9 | 89.8 | 739.1 | 197 |
| 231 |  | 115.39047 | 25.15352 | 2.4808 | 15.2700 | 7.1208 | 15.1532 | 2.6868 | 2.9125 | 0.0211 | 77.8 | 85.2 | 844.5 | 256.8 |
| 232 |  | 115.39455 | 25.15898 | 2.3816 | 15.9316 | 7.1129 | 13.3830 | 2.9698 | 3.8905 | 0.0170 | 100.5 | 128.3 | 1197.9 | 297.6 |
| 233 |  | 115.39478 | 25.15139 | 17.3228 | 15.1713 | 7.0481 | 11.9490 | 2.9336 | 3.4638 | 0.0239 | 97.8 | 116.1 | 988.3 | 278.8 |
| 234 |  | 115.38627 | 25.16452 | 2.8399 | 13.8045 | 8.6034 | 11.3694 | 3.1048 | 4.9728 | 0.0303 | 87.4 | 112.5 | 941.6 | 171.7 |
| 235 |  | 115.37189 | 25.13679 | 2.0821 | 12.4771 | 5.7597 | 8.1490 | 2.5448 | 2.6839 | 0.0184 | 81.2 | 83.9 | 895.5 | 198.9 |
| 236 |  | 115.44523 | 25.13453 | 1.8178 | 16.0703 | 7.5668 | 11.2351 | 2.4809 | 3.1510 | 0.0243 | 66.7 | 68.4 | 780.6 | 206.9 |
| 237 |  | 115.39380 | 25.14950 | 1.8014 | 16.2507 | 7.2695 | 10.0310 | 3.1768 | 3.2742 | 0.0225 | 93.9 | 103.1 | 1025.9 | 223.7 |
| 238 |  | 115.37524 | 25.16419 | 11.5229 | 15.8203 | 7.4165 | 11.0762 | 2.9682 | 3.6051 | 0.0340 | 94.8 | 132.9 | 1145.1 | 239.6 |
| 239 |  | 115.37086 | 25.16863 | 7.9541 | 12.7199 | 6.4454 | 8.6807 | 2.7854 | 2.7524 | 0.0376 | 88.7 | 100.2 | 989.1 | 223.9 |
| 240 |  | 115.40129 | 25.16644 | 1.8609 | 13.9493 | 8.0425 | 9.7628 | 2.3483 | 3.1332 | 0.0155 | 68.1 | 91 | 820 | 307.4 |
| 241 |  | 115.38642 | 25.19033 | 7.5622 | 15.6845 | 8.7716 | 17.5024 | 2.9501 | 3.1948 | 0.0297 | 91.8 | 104.3 | 1019.1 | 226.8 |
| 242 |  | 115.38659 | 25.18937 | 1.7270 | 13.3978 | 5.8553 | 8.6368 | 2.4272 | 2.6928 | 0.0305 | 82.2 | 103.2 | 1001 | 242.5 |
| 243 |  | 115.39486 | 25.18561 | 1.6077 | 15.7076 | 7.1931 | 19.3659 | 3.0543 | 3.6401 | 0.0252 | 92.4 | 123.3 | 1018.9 | 231.6 |
| 244 |  | 115.38215 | 25.16178 | 2.0099 | 13.2610 | 7.5717 | 8.2352 | 2.2743 | 2.7715 | 0.0339 | 83.8 | 104.6 | 923.5 | 153.9 |
| 245 |  | 115.36706 | 25.16044 | 2.1733 | 14.6677 | 6.6383 | 9.0160 | 2.0241 | 2.6648 | 0.0082 | 70.1 | 83.7 | 885.5 | 171.7 |
| 246 |  | 115.39832 | 25.16615 | 2.5000 | 16.4808 | 5.9887 | 10.7301 | 2.4372 | 3.3089 | 0.0294 | 94.7 | 126.5 | 1144.6 | 212.2 |
| 247 |  | 114.71393 | 25.70992 | 1.8652 | 15.8972 | 8.8417 | 9.9308 | 2.6923 | 3.7830 | 0.0244 | 91.5 | 116.6 | 1107.4 | 253.2 |
| 248 | Longnan City | 114.76017 | 24.85967 | 3.1472 | 16.8601 | 2.9132 | 11.2866 | 2.0991 | 3.7102 | 0.0104 | 74.7 | 126.8 | 1071.3 | 210.1 |
| 249 |  | 114.79747 | 24.65752 | 4.0896 | 16.2101 | 4.7970 | 10.8333 | 3.3626 | 3.3800 | 0.0168 | 101.7 | 150.4 | 1299.7 | 341.3 |
| 250 |  | 114.73697 | 24.85852 | 10.9873 | 28.2071 | 3.2484 | 14.6064 | 3.5905 | 4.8998 | 0.0268 | 109.7 | 133.2 | 1176.1 | 227.5 |
| 251 |  | 114.74115 | 24.86789 | 3.3165 | 30.3478 | 4.1682 | 13.3984 | 2.8481 | 4.0547 | 0.0260 | 113.8 | 121.5 | 1054.4 | 240.1 |
| 252 |  | 114.73670 | 24.87388 | 4.2268 | 27.8177 | 6.3577 | 10.7892 | 1.7336 | 3.3330 | 0.0144 | 102.9 | 118.8 | 1015.9 | 156.8 |
| 253 |  | 114.72618 | 24.87300 | 2.2294 | 22.3732 | 13.0929 | 28.3864 | 1.9976 | 4.5119 | 0.0311 | 119.9 | 434 | 1716.4 | 492.6 |
| 254 |  | 114.68355 | 24.83135 | 14.5589 | 19.5513 | 2.9976 | 12.5166 | 2.3545 | 3.4022 | 0.0192 | 82.3 | 138.7 | 1156.9 | 267.1 |
| 255 |  | 114.70756 | 24.81567 | 2.5206 | 18.2902 | 4.1230 | 11.2349 | 1.6341 | 3.2899 | 0.0167 | 87.3 | 136.7 | 1184 | 186.6 |
| 256 |  | 114.67609 | 24.80736 | 2.3779 | 13.4861 | 3.6777 | 12.9444 | 2.3880 | 3.8114 | 0.0144 | 85.1 | 110.8 | 1055.9 | 214.3 |
| 257 |  | 114.63014 | 24.79381 | 3.1256 | 20.4990 | 3.9787 | 12.5761 | 2.1569 | 5.0358 | 0.0256 | 87.3 | 143.2 | 1118.1 | 157 |
| 258 |  | 114.68531 | 24.83090 | 2.3689 | 17.9157 | 7.9321 | 13.6659 | 2.6783 | 3.6941 | 0.0388 | 109.4 | 142.9 | 1499.5 | 329.4 |
| 259 | Dingnan County | 114.92354 | 24.89733 | 4.3895 | 16.3623 | 2.8778 | 12.5765 | 1.9204 | 3.3275 | 0.0136 | 85.3 | 109.8 | 1156.2 | 181.4 |
| 260 |  | 115.32160 | 24.80524 | 5.1348 | 13.1842 | 3.1167 | 11.8670 | 3.6920 | 4.5883 | 0.0148 | 81.2 | 104.2 | 1094.4 | 233.4 |
| 261 |  | 115.23729 | 24.84599 | 2.0047 | 15.6114 | 5.8316 | 10.8721 | 3.0384 | 3.4064 | 0.0159 | 83.5 | 107.4 | 1052.5 | 239 |
| 262 |  | 115.25151 | 24.85317 | 3.9374 | 10.4241 | 2.6921 | 9.5690 | 2.4738 | 3.6259 | 0.0251 | 80.2 | 124.8 | 1011.7 | 320.1 |
| 263 |  | 115.25483 | 24.81250 | 13.7895 | 13.7077 | 3.1422 | 11.8794 | 2.5376 | 4.1210 | 0.0221 | 88.1 | 122.9 | 1136.5 | 203.5 |
| 264 |  | 115.22227 | 24.85615 | 1.0451 | 15.6126 | 2.7414 | 12.0970 | 3.7995 | 3.9244 | 0.0163 | 68.3 | 96.3 | 1116.5 | 215.8 |
| 265 |  | 115.02048 | 24.77315 | 3.3243 | 14.9035 | 1.7273 | 10.2400 | 2.0216 | 3.0814 | 0.0104 | 65.1 | 87.8 | 1152.6 | 180.8 |
| 266 |  | 115.20860 | 24.87485 | 2.5242 | 10.8897 | 5.4177 | 7.8718 | 2.3881 | 2.5774 | 0.0348 | 85.8 | 121.2 | 966.5 | 171.8 |
| 267 |  | 115.23141 | 24.88300 | 2.9180 | 12.4375 | 6.5798 | 10.3769 | 2.5260 | 2.9909 | 0.0411 | 93.1 | 139.8 | 1155.1 | 203.5 |
| 268 |  | 115.03243 | 24.88430 | 5.5862 | 11.0836 | 6.1669 | 7.9763 | 2.3405 | 2.5891 | 0.0174 | 76.7 | 106 | 887.7 | 198 |
| 269 |  | 115.14693 | 24.73430 | 4.3256 | 11.1041 | 5.5558 | 9.6722 | 2.3085 | 3.9583 | 0.0268 | 81.3 | 107.3 | 1093.1 | 173.7 |
| 270 |  | 115.01955 | 24.67969 | 2.5764 | 14.2061 | 6.9817 | 14.3202 | 2.6869 | 2.7400 | 0.0276 | 84.3 | 118.2 | 1023.3 | 154.6 |
| 271 | Quanan County | 114.56352 | 24.95505 | 4.0430 | 16.9922 | 5.3485 | 13.6007 | 3.0950 | 4.4155 | 0.0156 | 129.1 | 115.8 | 1347.3 | 306.4 |
| 272 |  | 114.56853 | 24.91586 | 2.8506 | 13.5876 | 20.7734 | 12.1745 | 2.8677 | 4.3640 | 0.0176 | 102 | 121.4 | 1003.5 | 198.3 |
| 273 |  | 114.53801 | 24.97872 | 2.1622 | 10.4423 | 7.0585 | 10.2267 | 1.9609 | 4.5865 | 0.0127 | 90.2 | 79.2 | 847.9 | 180.7 |
| 274 |  | 114.54715 | 24.96065 | 17.2246 | 12.3950 | 24.2104 | 13.0510 | 3.3736 | 5.1518 | 0.0157 | 119.8 | 134.8 | 1153.9 | 201.3 |
| 275 |  | 114.54146 | 24.96282 | 2.7590 | 21.2259 | 13.5782 | 10.5925 | 2.5895 | 3.9681 | 0.0361 | 97.3 | 122.3 | 770 | 337.8 |
| 276 |  | 114.59038 | 25.03775 | 7.4625 | 22.5013 | 5.9989 | 14.2552 | 2.1958 | 3.9793 | 0.0299 | 98.3 | 124.9 | 1103.4 | 235.2 |
| 277 |  | 114.39502 | 24.73907 | 3.3077 | 14.4231 | 6.5037 | 8.7019 | 2.4012 | 2.6831 | 0.0285 | 87.3 | 112.7 | 952.7 | 199.5 |
| 278 |  | 114.39257 | 24.76046 | 2.3591 | 15.3531 | 7.9247 | 9.9471 | 2.0188 | 2.8337 | 0.0172 | 73 | 86.7 | 841.8 | 258.8 |
| 279 |  | 114.53954 | 24.81811 | 3.6467 | 14.0263 | 7.8253 | 9.6040 | 2.0338 | 2.6219 | 0.0257 | 82.5 | 99.9 | 963.6 | 272.4 |
| 280 |  | 114.41565 | 24.72585 | 2.3132 | 18.6353 | 6.1123 | 12.6460 | 2.1419 | 6.8415 | 0.0239 | 109 | 117.8 | 1354.1 | 434 |
| 281 |  | 114.39495 | 24.64036 | 2.6376 | 13.3861 | 3.5801 | 9.9377 | 2.5655 | 3.8003 | 0.0192 | 82.1 | 129.7 | 1227 | 208.1 |
| 282 |  | 114.37052 | 24.70196 | 1.6856 | 13.6020 | 8.1909 | 10.8912 | 2.0288 | 3.6014 | 0.0229 | 80.7 | 100.6 | 1000.4 | 315.9 |
| 283 | Xingguo County  Xingguo County | 115.33650 | 26.26775 | 4.4996 | 16.7012 | 2.8273 | 11.7053 | 1.9581 | 3.6404 | 0.0315 | 88.5 | 120.4 | 925.6 | 280 |
| 284 |  | 115.30226 | 26.41582 | 5.0306 | 13.6313 | 2.4176 | 9.3603 | 2.6954 | 3.5889 | 0.0205 | 73.7 | 141.3 | 896.2 | 331.3 |
| 285 |  | 115.14771 | 26.33481 | 3.2783 | 19.8598 | 4.7794 | 10.6480 | 2.5806 | 3.9436 | 0.0397 | 113.3 | 140.6 | 1169.6 | 270.5 |
| 286 |  | 115.29830 | 26.48876 | 7.8893 | 22.6127 | 2.6440 | 13.3570 | 1.6312 | 3.4870 | 0.0774 | 94.7 | 140.4 | 1086.1 | 147.2 |
| 287 |  | 115.40195 | 26.50486 | 1.5839 | 20.6213 | 3.0248 | 13.6739 | 2.8194 | 3.7241 | 0.0165 | 77.8 | 140.9 | 1289.4 | 242.1 |
| 288 |  | 115.38677 | 26.21532 | 24.6760 | 20.7709 | 3.1742 | 11.8557 | 3.1153 | 3.8623 | 0.0187 | 96.1 | 158.7 | 1235.8 | 245.7 |
| 289 |  | 115.38869 | 26.48996 | 3.3240 | 19.9295 | 4.1646 | 9.7654 | 2.0822 | 2.6358 | 0.0131 | 67.2 | 96 | 781.7 | 223.2 |
| 290 |  | 115.36064 | 26.56414 | 3.3890 | 20.2767 | 5.1056 | 12.5456 | 2.7529 | 3.3705 | 0.0151 | 72.8 | 137.9 | 1113.5 | 178.8 |
| 291 |  | 115.08933 | 26.40366 | 4.9776 | 20.8077 | 3.0567 | 12.0108 | 2.5133 | 3.8240 | 0.0196 | 104.6 | 115 | 1132.9 | 260.5 |
| 292 |  | 115.34735 | 26.47994 | 4.6051 | 20.9750 | 5.6330 | 11.6394 | 2.3223 | 4.0735 | 0.0201 | 99.7 | 89.7 | 852.3 | 269 |
| 293 |  | 115.34292 | 26.27397 | 3.9260 | 22.6227 | 2.4176 | 16.4880 | 1.9717 | 3.0946 | 0.0319 | 99.5 | 113 | 1320.1 | 289 |
| 294 |  | 115.25295 | 26.29062 | 3.4924 | 24.2122 | 4.8398 | 14.6651 | 3.0875 | 4.6370 | 0.0166 | 124.4 | 146.1 | 1130.1 | 249.3 |
| 295 |  | 115.34836 | 26.52631 | 4.6038 | 20.2235 | 10.1790 | 13.3945 | 2.9139 | 3.5673 | 0.0386 | 138.7 | 109.8 | 1322.4 | 191.7 |
| 296 |  | 115.07258 | 26.37685 | 3.6582 | 15.8074 | 3.9403 | 10.2120 | 3.0738 | 3.3621 | 0.0185 | 83.6 | 110.1 | 1056.8 | 193.7 |
| 297 |  | 115.09893 | 26.32623 | 3.2578 | 16.6615 | 3.7146 | 12.8927 | 3.2765 | 4.0236 | 0.0317 | 109.3 | 123.8 | 1099.8 | 259.3 |
| 298 |  | 115.41339 | 26.23017 | 2.9038 | 13.0377 | 3.1840 | 15.8441 | 2.3693 | 2.7948 | 0.0308 | 123.9 | 221.3 | 1605.2 | 294.1 |
| 299 |  | 115.35241 | 26.53367 | 16.0378 | 19.1552 | 10.3633 | 13.2365 | 3.3847 | 3.6334 | 0.0199 | 113.9 | 181.2 | 1430.9 | 192.4 |
| 300 |  | 115.28152 | 26.46949 | 4.5159 | 15.2910 | 1.0832 | 13.2018 | 2.9313 | 3.5382 | 0.0183 | 82.3 | 180.4 | 1829.1 | 198 |
| 301 |  | 115.31506 | 26.48080 | 3.7614 | 16.0622 | 2.2187 | 12.2908 | 2.2312 | 2.8002 | 0.0139 | 86.5 | 173.5 | 1607.4 | 183.2 |
| 302 |  | 115.34259 | 26.28404 | 6.4881 | 18.7676 | 4.2610 | 11.8961 | 3.0074 | 4.0448 | 0.0123 | 83.9 | 144.8 | 1274.6 | 229.5 |
| 303 |  | 115.40195 | 26.50486 | 3.1934 | 15.8557 | 2.8825 | 12.6234 | 2.4492 | 4.2108 | 0.0155 | 77.3 | 135 | 1219.4 | 153.7 |
| 304 |  | 115.49464 | 26.50577 | 3.7893 | 12.0858 | 3.0454 | 9.0248 | 2.1395 | 2.8856 | 0.0188 | 63.7 | 118.8 | 1008.4 | 231.7 |
| 305 |  | 115.24005 | 26.47701 | 6.2753 | 17.2987 | 1.7269 | 11.8124 | 1.6503 | 2.9650 | 0.0171 | 92.8 | 96.9 | 1086.5 | 149.3 |
| 306 |  | 115.16169 | 26.33387 | 2.9270 | 19.8129 | 29.6595 | 13.2779 | 3.1156 | 3.9210 | 0.0164 | 105.9 | 130.1 | 1208.4 | 224.9 |
| 307 |  | 115.49106 | 26.50695 | 2.5820 | 17.8285 | 21.1348 | 13.2619 | 2.4116 | 3.8749 | 0.0153 | 118 | 142.4 | 1322.6 | 271.9 |
| 308 | Ningdu County  Ningdu County  Ningdu County | 116.12913 | 26.25903 | 2.4404 | 13.6754 | 9.3074 | 10.0370 | 1.8547 | 3.4253 | 0.0148 | 82 | 142.3 | 1060 | 190.8 |
| 309 |  | 116.11843 | 26.24953 | 2.4061 | 17.7723 | 10.0505 | 15.2051 | 2.7895 | 5.9060 | 0.0335 | 111.2 | 201.8 | 1493.4 | 262.2 |
| 310 |  | 116.12897 | 26.26342 | 1.8600 | 18.2047 | 3.4980 | 13.0780 | 1.6378 | 4.0404 | 0.0233 | 97.2 | 98.3 | 1079.4 | 288.1 |
| 311 |  | 116.11914 | 26.24971 | 2.5142 | 16.7196 | 8.4619 | 12.6347 | 2.6010 | 4.4912 | 0.0231 | 102.7 | 151.6 | 1188.8 | 270.1 |
| 312 |  | 116.11791 | 26.24742 | 2.2550 | 14.3335 | 3.4900 | 10.1794 | 2.4119 | 3.0015 | 0.0129 | 88.5 | 104.2 | 1246.1 | 236 |
| 313 |  | 116.11833 | 26.24882 | 2.2811 | 18.1910 | 6.2885 | 12.0361 | 2.5917 | 4.0277 | 0.0210 | 102.3 | 163.8 | 1164.4 | 317.3 |
| 314 |  | 116.10846 | 26.27296 | 2.5440 | 16.4489 | 7.4388 | 10.1562 | 3.3788 | 4.1029 | 0.0168 | 73.9 | 114.5 | 1024.1 | 288.5 |
| 315 |  | 116.15804 | 26.22868 | 2.3617 | 13.6835 | 7.1451 | 9.9377 | 2.2197 | 4.7864 | 0.0135 | 84.8 | 122.4 | 1025.9 | 198 |
| 316 |  | 116.01373 | 26.42552 | 2.2062 | 19.7867 | 5.4388 | 10.3268 | 1.5471 | 2.9974 | 0.0090 | 80.4 | 139.4 | 1192.7 | 253 |
| 317 |  | 115.96651 | 26.48231 | 2.2753 | 13.4122 | 3.6950 | 11.6176 | 2.5423 | 3.7386 | 0.0045 | 95.8 | 120.3 | 1307.2 | 179 |
| 318 |  | 116.09587 | 26.45256 | 1.6741 | 21.9466 | 7.7374 | 11.3645 | 3.2133 | 3.9781 | 0.0162 | 94.2 | 261.3 | 1384.7 | 399.3 |
| 319 |  | 116.14705 | 26.23677 | 15.8263 | 15.6922 | 4.2871 | 8.9305 | 2.0551 | 5.7394 | 0.0232 | 85.5 | 166 | 1036.7 | 371.9 |
| 320 |  | 115.85531 | 26.43763 | 3.9703 | 17.1829 | 7.7298 | 10.6657 | 2.5229 | 6.0179 | 0.0178 | 95.5 | 155 | 1246.5 | 191.1 |
| 321 |  | 115.87981 | 26.46088 | 5.9498 | 15.2483 | 3.9183 | 10.5634 | 3.4210 | 3.6549 | 0.0198 | 100.3 | 129.2 | 1366.8 | 328.3 |
| 322 |  | 115.88530 | 26.46359 | 2.9462 | 15.8010 | 6.4176 | 14.8272 | 2.8058 | 3.2221 | 0.0193 | 112 | 145.6 | 1337.3 | 314.8 |
| 323 |  | 115.88518 | 26.45854 | 2.1783 | 15.1369 | 9.5982 | 9.3059 | 2.3938 | 4.0895 | 0.0046 | 90.4 | 143 | 1204.4 | 252.1 |
| 324 |  | 115.84863 | 26.42908 | 16.1108 | 13.6426 | 3.6588 | 11.1755 | 2.9823 | 4.4729 | 0.0073 | 103.7 | 159.8 | 1547.3 | 251.6 |
| 325 |  | 116.19239 | 26.30983 | 9.3898 | 17.7300 | 6.1894 | 18.3196 | 2.2595 | 4.5258 | 0.0272 | 91.9 | 118.7 | 1028.6 | 264.3 |
| 326 |  | 115.83809 | 26.43195 | 4.2159 | 16.1077 | 7.4986 | 12.6333 | 2.6326 | 3.7598 | 0.0152 | 88.8 | 133.2 | 1172.7 | 263.7 |
| 327 |  | 115.86514 | 26.43201 | 9.5873 | 16.4370 | 5.4309 | 10.8460 | 3.1045 | 4.1735 | 0.0097 | 105.7 | 121.5 | 1372.1 | 215.5 |
| 328 |  | 115.88146 | 26.45795 | 8.0759 | 14.1882 | 3.0663 | 9.0630 | 2.0815 | 2.6635 | 0.0077 | 90.8 | 130.6 | 1397.6 | 281.7 |
| 329 |  | 115.88721 | 26.45595 | 2.0647 | 14.7045 | 9.6783 | 14.8363 | 2.4295 | 4.7670 | 0.0132 | 89 | 145.6 | 1303.9 | 346.7 |
| 330 |  | 115.88093 | 26.46140 | 3.1921 | 16.2671 | 2.4055 | 10.6959 | 2.5615 | 2.9539 | 0.0276 | 89.7 | 125.5 | 997 | 146.7 |
| 331 |  | 115.86729 | 26.43627 | 3.7346 | 15.6321 | 3.3524 | 11.0434 | 3.1154 | 4.4942 | 0.0217 | 105.3 | 144.3 | 1402.1 | 224.4 |
| 332 |  | 116.10820 | 26.56074 | 2.5980 | 13.9653 | 2.1496 | 11.1529 | 3.0379 | 3.2081 | 0.0246 | 88.2 | 129.5 | 1019.6 | 155.9 |
| 333 |  | 115.69380 | 26.58994 | 3.7444 | 16.5456 | 4.7512 | 8.2386 | 1.5470 | 3.1208 | 0.0087 | 77.3 | 112.1 | 1038 | 211.7 |
| 334 |  | 115.85885 | 26.44961 | 3.3294 | 13.9311 | 4.9305 | 9.4542 | 2.4167 | 3.4976 | 0.0163 | 84.1 | 111 | 1276.7 | 243.4 |
| 335 |  | 115.87783 | 26.46076 | 1.8196 | 13.7112 | 2.9528 | 8.5120 | 2.4771 | 3.2331 | 0.0145 | 77.2 | 123.9 | 820 | 336.1 |
| 336 |  | 116.01001 | 26.47704 | 2.8116 | 12.7052 | 2.1771 | 9.9087 | 2.3361 | 3.3404 | 0.0113 | 76.3 | 151.7 | 1156.7 | 272.7 |
| 337 |  | 115.88764 | 26.45844 | 9.3257 | 13.3650 | 2.9450 | 9.9719 | 2.4678 | 2.9977 | 0.0130 | 72.8 | 122.7 | 938.6 | 144.7 |
| 338 |  | 115.85683 | 26.44522 | 4.6745 | 13.0428 | 2.0830 | 9.9386 | 3.1232 | 3.6384 | 0.0216 | 76.3 | 111 | 1001.9 | 202.1 |
| 339 |  | 115.85885 | 26.44961 | 13.8418 | 11.1234 | 2.9037 | 9.3766 | 2.2639 | 3.4712 | 0.0142 | 79.5 | 104 | 1103 | 241.4 |
| 340 |  | 115.70079 | 26.34857 | 5.3537 | 15.2883 | 6.4559 | 9.8356 | 2.2872 | 3.8050 | 0.0201 | 100.1 | 121 | 1177.4 | 225 |
| 341 |  | 115.74122 | 26.03264 | 3.8580 | 14.4097 | 3.2301 | 8.4207 | 2.5530 | 3.9608 | 0.0229 | 88.7 | 133.8 | 1214.8 | 280.1 |
| 342 |  | 115.90496 | 26.43912 | 4.7328 | 15.1390 | 4.7947 | 9.6437 | 2.9016 | 4.5091 | 0.0125 | 86.5 | 150.6 | 1285.2 | 162.5 |
| 343 |  | 115.82790 | 26.43644 | 5.7875 | 17.9059 | 3.4653 | 21.4612 | 2.5844 | 3.3614 | 0.0193 | 81.3 | 100.4 | 1166.8 | 246.9 |
| 344 |  | 115.89834 | 26.43911 | 10.8594 | 18.2557 | 7.6543 | 10.3625 | 2.7308 | 4.0869 | 0.0162 | 90.7 | 116.9 | 1039.2 | 248 |
| 345 |  | 115.80903 | 26.49212 | 2.4886 | 19.8712 | 4.2851 | 9.6245 | 2.9584 | 3.6516 | 0.0155 | 87 | 119.7 | 1090.7 | 267.2 |
| 346 |  | 115.87867 | 26.45745 | 3.4215 | 15.5940 | 4.0953 | 18.9413 | 2.3996 | 3.9179 | 0.0626 | 86.9 | 118.5 | 1098 | 227.9 |
| 347 |  | 115.73048 | 25.98068 | 4.6881 | 15.0928 | 25.3715 | 11.0212 | 3.1931 | 3.8673 | 0.0134 | 92.6 | 116.5 | 1035.5 | 241.1 |
| 348 |  | 115.87702 | 26.46070 | 8.3390 | 17.7325 | 1.6863 | 8.6285 | 2.4408 | 2.9763 | 0.0205 | 84.6 | 145 | 1093 | 304.3 |
| 349 |  | 115.88421 | 26.45540 | 32.5888 | 12.8176 | 1.6819 | 7.6818 | 2.6727 | 2.4685 | 0.0205 | 110.8 | 123.4 | 1362.7 | 269.6 |
| 350 |  | 115.89123 | 26.45648 | 3.4390 | 18.1881 | 4.7102 | 10.8835 | 2.2315 | 3.5244 | 0.0156 | 83 | 108.8 | 987.2 | 261.1 |
| 351 |  | 115.89140 | 26.46126 | 7.7041 | 17.3929 | 14.8128 | 10.3694 | 2.9572 | 2.8343 | 0.0201 | 76.6 | 133.5 | 1426.2 | 185.7 |
| 352 |  | 115.85467 | 26.30648 | 3.4016 | 15.4083 | 11.4600 | 11.3289 | 2.7718 | 3.4872 | 0.0129 | 84 | 128.9 | 919.5 | 186.4 |
| 353 |  | 115.89038 | 26.46102 | 6.2654 | 16.3270 | 1.9831 | 9.4027 | 2.9758 | 2.8181 | 0.0192 | 77.3 | 117.4 | 1392.9 | 140 |
| 354 |  | 115.89035 | 26.43587 | 3.2914 | 13.5682 | 3.6532 | 9.2030 | 2.6753 | 2.8518 | 0.0151 | 92.1 | 112.3 | 1073.8 | 218.8 |
| 355 |  | 115.86685 | 26.39929 | 3.2736 | 15.3391 | 4.8918 | 9.1076 | 2.3755 | 3.0577 | 0.0179 | 73.7 | 106 | 988.6 | 203.4 |
| 356 |  | 115.89187 | 26.45310 | 5.5333 | 12.9098 | 2.7754 | 8.4289 | 2.5848 | 3.0973 | 0.0266 | 96.3 | 151.8 | 1238.3 | 134.3 |
| 357 | Yudu County  Yudu County | 115.64513 | 25.90774 | 2.8049 | 19.0148 | 6.8906 | 10.4360 | 2.1094 | 4.4162 | 0.0117 | 103.5 | 121.8 | 1118.5 | 305.8 |
| 358 |  | 115.63324 | 25.90395 | 2.5843 | 19.8671 | 7.8435 | 12.2115 | 2.9534 | 4.2342 | 0.0213 | 94.1 | 120.8 | 1054.8 | 285.4 |
| 359 |  | 115.31936 | 25.88694 | 3.8659 | 13.6635 | 2.6010 | 12.3948 | 2.6867 | 3.0792 | 0.0133 | 84.4 | 135.7 | 1346.3 | 171 |
| 360 |  | 115.27479 | 25.86468 | 3.5845 | 16.5834 | 2.9160 | 15.0942 | 2.4122 | 4.2220 | 0.0267 | 99.8 | 144.6 | 1190.2 | 227.1 |
| 361 |  | 115.27220 | 25.82748 | 19.7153 | 18.3972 | 7.2185 | 11.8646 | 3.0146 | 4.3945 | 0.0109 | 123.7 | 156.4 | 1476.4 | 287.1 |
| 362 |  | 115.46008 | 25.73595 | 3.2701 | 17.6049 | 11.1780 | 14.3132 | 2.7973 | 4.4783 | 0.0243 | 128.5 | 120.3 | 1433.9 | 282.8 |
| 363 |  | 115.41984 | 25.65234 | 2.2001 | 26.6915 | 5.7676 | 14.0080 | 2.4703 | 4.2686 | 0.0152 | 98.8 | 142.8 | 1155.2 | 279.6 |
| 364 |  | 115.31779 | 25.78710 | 2.1744 | 26.0577 | 7.5199 | 12.5039 | 2.4740 | 5.4198 | 0.0221 | 80.8 | 123.3 | 973.7 | 232 |
| 365 |  | 115.32024 | 25.76966 | 13.6676 | 20.2765 | 6.8436 | 14.3136 | 2.3600 | 3.1456 | 0.0191 | 97.8 | 138.3 | 1204.2 | 239.6 |
| 366 |  | 115.27063 | 25.84436 | 4.1595 | 14.1997 | 14.9124 | 14.9692 | 3.2397 | 4.0122 | 0.0417 | 95 | 136.8 | 973 | 182.3 |
| 367 |  | 115.54947 | 25.91613 | 3.1657 | 15.0584 | 11.5318 | 13.1527 | 2.7138 | 3.3972 | 0.0249 | 99.1 | 87.9 | 1079.9 | 209.8 |
| 368 |  | 115.45520 | 25.99486 | 2.2879 | 10.9615 | 4.0143 | 16.8061 | 1.2469 | 2.9339 | 0.0322 | 89 | 141.3 | 1340.6 | 146.3 |
| 369 |  | 115.54201 | 25.61755 | 3.4813 | 16.8496 | 14.2757 | 15.0039 | 1.8952 | 3.2571 | 0.0221 | 91.8 | 135.2 | 1291.1 | 198 |
| 370 |  | 115.55811 | 25.72957 | 2.6543 | 17.0256 | 9.1374 | 19.9402 | 1.5602 | 3.3170 | 0.0920 | 108.1 | 164.8 | 1382.6 | 289.5 |
| 371 |  | 115.53224 | 25.81026 | 2.8599 | 17.5966 | 5.7461 | 11.7392 | 2.5180 | 2.6541 | 0.0233 | 87.7 | 105.7 | 907.8 | 251.8 |
| 372 |  | 115.25795 | 25.91493 | 7.1485 | 16.5517 | 2.9734 | 11.5328 | 3.2170 | 3.8687 | 0.0084 | 81.4 | 100.3 | 942.1 | 286.7 |
| 373 |  | 115.27199 | 25.93586 | 4.1389 | 14.5957 | 2.4770 | 9.4842 | 2.2252 | 3.2091 | 0.0209 | 151 | 133.7 | 782.7 | 487.6 |
| 374 |  | 115.30317 | 25.93712 | 5.9114 | 16.9943 | 2.8575 | 11.2319 | 3.4692 | 4.3612 | 0.0101 | 93.3 | 170.9 | 1339 | 330.1 |
| 375 |  | 115.24915 | 25.89530 | 8.8860 | 13.5799 | 2.1643 | 9.1332 | 2.8519 | 2.7894 | 0.0275 | 93.1 | 126.4 | 1086.7 | 195.7 |
| 376 |  | 115.48610 | 25.80208 | 5.6661 | 16.4330 | 27.3954 | 11.3474 | 3.0366 | 3.8603 | 0.0234 | 95.4 | 114.9 | 1052.8 | 237 |
| 377 |  | 115.48869 | 25.79434 | 6.5035 | 12.2436 | 2.1150 | 8.4528 | 2.4962 | 2.5374 | 0.0167 | 93.1 | 119.3 | 1037.2 | 220.4 |
| 378 |  | 115.38829 | 25.84950 | 12.3602 | 13.2988 | 2.3270 | 12.7937 | 3.2222 | 2.8695 | 0.0191 | 113.9 | 187.4 | 1791.9 | 207.1 |
| 379 |  | 115.50798 | 25.74683 | 14.6487 | 13.1105 | 2.4866 | 9.8826 | 2.1047 | 2.2624 | 0.0226 | 110.7 | 127.6 | 1106.3 | 246 |
| 380 |  | 115.55857 | 25.73628 | 4.6166 | 15.7000 | 3.9430 | 12.4631 | 2.5914 | 3.6873 | 0.0165 | 80.5 | 116.3 | 976.8 | 201.6 |
| 381 |  | 115.49792 | 25.81047 | 7.0847 | 16.3349 | 2.3625 | 10.1895 | 2.3185 | 3.2880 | 0.0354 | 83.4 | 134.7 | 936.5 | 274.2 |
| 382 |  | 115.52235 | 25.84117 | 3.3884 | 12.6380 | 1.9756 | 8.4393 | 2.6976 | 2.9927 | 0.0215 | 93.9 | 124.9 | 1200.3 | 139.9 |
| 383 |  | 115.48642 | 25.86354 | 4.7788 | 15.4523 | 6.3157 | 10.0128 | 2.9569 | 4.2382 | 0.0271 | 79 | 119.4 | 1202 | 217.7 |
| 384 |  | 115.31222 | 25.95297 | 5.1879 | 14.5141 | 1.8490 | 8.4300 | 2.9946 | 2.7486 | 0.0181 | 72.1 | 110.5 | 975.7 | 206.4 |
| 385 |  | 115.38581 | 26.00702 | 2.4523 | 16.2095 | 2.9931 | 9.6426 | 2.1371 | 2.9771 | 0.0143 | 93 | 128 | 1065.6 | 257.4 |
| 386 |  | 115.62318 | 25.90421 | 6.8119 | 15.1026 | 4.5642 | 8.8493 | 2.3584 | 3.2494 | 0.0194 | 106.3 | 133.4 | 1144.7 | 490.2 |
| 387 |  | 115.58811 | 25.91851 | 5.5812 | 11.9215 | 3.7324 | 9.8284 | 2.5785 | 2.9387 | 0.0126 | 74.6 | 143.2 | 1155.8 | 176.8 |
| 388 |  | 115.50750 | 25.94525 | 3.3743 | 16.0772 | 2.9529 | 9.1413 | 2.3659 | 3.8220 | 0.0144 | 73.3 | 87.7 | 1031.4 | 185.7 |
| 389 |  | 115.56327 | 25.92237 | 5.3314 | 11.9638 | 2.7960 | 8.7120 | 2.7130 | 2.7039 | 0.0182 | 99.2 | 119.5 | 1110.2 | 232.4 |
| 390 |  | 115.79427 | 26.00048 | 5.5494 | 12.8073 | 2.2495 | 9.6209 | 2.6919 | 2.9744 | 0.0231 | 110.1 | 127.9 | 1296 | 265.5 |
| 391 |  | 115.78018 | 25.98840 | 3.9947 | 11.9652 | 3.1548 | 8.8662 | 2.8054 | 2.9134 | 0.0239 | 110.4 | 178.5 | 1421.9 | 218.2 |
| 392 |  | 115.61722 | 26.29857 | 8.2524 | 12.4170 | 2.5362 | 9.4012 | 2.3853 | 2.4068 | 0.0150 | 86.2 | 116.6 | 1155.3 | 205.2 |
| 393 |  | 115.57081 | 26.24155 | 3.0993 | 19.5781 | 12.5935 | 11.3837 | 2.8155 | 3.9941 | 0.0134 | 110.4 | 117 | 1360.3 | 262.5 |
| 394 |  | 115.67836 | 26.00372 | 10.0984 | 13.7553 | 2.4870 | 10.2250 | 3.1106 | 2.9744 | 0.0160 | 91.3 | 131.6 | 1143.7 | 184.5 |
| 395 |  | 115.65220 | 25.99567 | 7.0717 | 13.8668 | 1.9195 | 9.6346 | 3.4180 | 3.2464 | 0.0220 | 95.1 | 145.8 | 1397.5 | 207 |
| 396 |  | 115.39979 | 25.84612 | 4.5899 | 12.3715 | 1.8940 | 9.3830 | 2.9432 | 2.8651 | 0.0270 | 110.7 | 174.3 | 1438.5 | 222.8 |
| 397 |  | 115.41043 | 25.83467 | 5.0233 | 13.3057 | 14.2641 | 10.7983 | 2.2915 | 3.0957 | 0.0199 | 91.7 | 125 | 1001.9 | 221.8 |
| 398 | Ruijin City  Ruijin City  Ruijin City | 115.96882 | 25.92857 | 6.5801 | 19.0442 | 2.5278 | 12.1957 | 2.5571 | 4.1453 | 0.1814 | 81.4 | 136.5 | 1242.4 | 168 |
| 399 |  | 115.97697 | 25.85640 | 2.1457 | 16.0843 | 3.4088 | 10.6686 | 2.7888 | 3.4821 | 0.1091 | 62.9 | 100.6 | 931.3 | 200.8 |
| 400 |  | 115.85147 | 25.66741 | 1.4531 | 15.6912 | 6.6790 | 12.4437 | 3.2822 | 4.6180 | 0.0786 | 88.2 | 149.1 | 1114 | 236.1 |
| 401 |  | 115.90664 | 25.89216 | 3.5343 | 17.2026 | 2.4809 | 12.8416 | 2.2482 | 3.8234 | 0.0656 | 71 | 119.7 | 1086.3 | 133.2 |
| 402 |  | 115.88326 | 25.94583 | 8.0803 | 17.1950 | 3.1207 | 11.5557 | 3.1062 | 4.0549 | 0.0565 | 89.9 | 104.2 | 1028.4 | 140.8 |
| 403 |  | 115.89296 | 25.83794 | 7.1693 | 16.4312 | 1.8233 | 11.9652 | 2.9531 | 4.3038 | 0.0581 | 76.4 | 112.6 | 1052.4 | 158.9 |
| 404 |  | 115.89423 | 25.83807 | 4.6348 | 15.6355 | 2.4753 | 12.1947 | 2.8420 | 3.2135 | 0.0370 | 81.9 | 118.5 | 1058.5 | 133 |
| 405 |  | 115.89163 | 25.83893 | 4.4698 | 18.5329 | 4.4402 | 11.5361 | 3.4742 | 3.3928 | 0.0469 | 87.4 | 134.5 | 1286.3 | 392 |
| 406 |  | 115.92963 | 25.75946 | 3.2015 | 18.0434 | 4.9098 | 11.5600 | 2.5107 | 4.0973 | 0.0361 | 88.2 | 125.3 | 1191.7 | 211.2 |
| 407 |  | 115.89111 | 25.84275 | 4.0005 | 17.3075 | 3.8149 | 10.0999 | 3.4088 | 4.9446 | 0.0334 | 79.3 | 119.2 | 1146.9 | 258.1 |
| 408 |  | 115.89810 | 25.81424 | 3.8807 | 15.0747 | 6.5021 | 12.0065 | 2.3066 | 4.1023 | 0.0307 | 85.6 | 128.7 | 1317.4 | 127.2 |
| 409 |  | 115.89631 | 25.80556 | 4.2866 | 16.7142 | 3.0453 | 11.7799 | 2.1840 | 3.4889 | 0.0634 | 87.9 | 139.7 | 1318.3 | 380 |
| 410 |  | 115.89370 | 25.84088 | 3.3981 | 15.3161 | 5.2240 | 10.9467 | 2.5213 | 3.2596 | 0.0461 | 85.7 | 137.4 | 1169.5 | 117.3 |
| 411 |  | 115.92647 | 25.79570 | 3.1045 | 17.5358 | 10.5688 | 14.9197 | 2.8795 | 4.6677 | 0.0590 | 98.4 | 134.6 | 1254.8 | 172.8 |
| 412 |  | 115.91983 | 25.85544 | 1.5188 | 17.0840 | 20.0064 | 15.8294 | 2.0749 | 3.8914 | 0.0309 | 86.6 | 106.1 | 1056.1 | 261.5 |
| 413 |  | 115.93884 | 25.75926 | 2.4423 | 19.6714 | 5.7677 | 12.3450 | 2.6289 | 3.9820 | 0.0368 | 81.8 | 128.5 | 1045.3 | 211.5 |
| 414 |  | 115.88065 | 25.69110 | 7.5884 | 20.9557 | 14.0720 | 69.0470 | 2.0785 | 3.3157 | 0.0174 | 92 | 84.3 | 1144.7 | 159.3 |
| 415 |  | 115.93985 | 25.75950 | 1.8055 | 15.4890 | 5.7113 | 10.2317 | 1.8289 | 4.2315 | 0.0211 | 65.8 | 105.8 | 1034.6 | 122.1 |
| 416 |  | 115.93848 | 25.76011 | 2.8481 | 13.6893 | 14.1311 | 12.2532 | 3.1492 | 4.2918 | 0.0425 | 106.7 | 129.3 | 1162.3 | 117.3 |
| 417 |  | 115.93856 | 25.75997 | 2.5198 | 17.4634 | 8.0715 | 11.7412 | 3.3439 | 5.5295 | 0.0254 | 88 | 106.2 | 1139.4 | 866.7 |
| 418 |  | 115.91767 | 25.69757 | 3.6709 | 17.7885 | 10.1973 | 12.9428 | 2.7233 | 5.3736 | 0.0161 | 86.8 | 74.3 | 1117.9 | 255.2 |
| 419 |  | 116.05291 | 25.97635 | 4.6832 | 17.5730 | 6.2323 | 13.6152 | 2.7451 | 5.3938 | 0.0372 | 73.2 | 84.2 | 973.8 | 215.4 |
| 420 |  | 116.04939 | 25.97396 | 20.6246 | 15.7582 | 4.1308 | 11.5037 | 2.0090 | 3.9933 | 0.0377 | 96.7 | 122.5 | 1270 | 207.6 |
| 421 |  | 116.04823 | 25.97284 | 2.3882 | 19.1924 | 2.3041 | 12.6203 | 2.1710 | 2.7811 | 0.0190 | 84.5 | 122.6 | 1205.9 | 227.8 |
| 422 |  | 116.05175 | 25.97286 | 3.0029 | 17.5160 | 2.6058 | 9.6726 | 2.1392 | 2.7470 | 0.0324 | 74.8 | 83.9 | 1037.8 | 226.7 |
| 423 |  | 116.05439 | 25.97245 | 3.4370 | 2.0206 | 0.0836 | 2.7433 | 0.2213 | 0.0062 | 0.0122 | 59.8 | 99.9 | 830.1 | 258 |
| 424 |  | 116.05758 | 25.97197 | 7.1523 | 18.2712 | 4.3570 | 12.6797 | 2.2191 | 3.2593 | 0.0248 | 69.3 | 82.5 | 968 | 262.6 |
| 425 |  | 116.05550 | 25.97749 | 11.6797 | 20.8662 | 7.9316 | 14.4705 | 2.9494 | 4.7097 | 0.0189 | 92 | 132.1 | 1232.2 | 294.2 |
| 426 |  | 116.05529 | 25.97440 | 3.3625 | 22.6677 | 3.6551 | 14.5121 | 3.0623 | 4.8854 | 0.0276 | 81.2 | 102.5 | 952 | 418.4 |
| 427 |  | 116.05207 | 25.98032 | 12.9064 | 19.1639 | 1.8506 | 12.7458 | 2.7937 | 3.9446 | 0.0448 | 64.8 | 97.3 | 893.8 | 232 |
| 428 |  | 116.04818 | 25.97795 | 10.8636 | 18.4063 | 2.8512 | 17.5408 | 3.0501 | 4.0995 | 0.0296 | 82.3 | 280.1 | 1209.4 | 494.8 |
| 429 |  | 116.04718 | 25.97679 | 2.0163 | 19.7174 | 7.7179 | 14.0349 | 2.4407 | 3.9723 | 0.0247 | 78.8 | 203.6 | 1078.9 | 336.2 |
| 430 |  | 116.05879 | 25.98211 | 3.6855 | 18.6499 | 2.4690 | 13.1032 | 1.9636 | 3.5254 | 0.0362 | 68.3 | 128.7 | 1062 | 242.2 |
| 431 |  | 116.06299 | 25.97681 | 8.1868 | 17.5975 | 2.9632 | 10.0893 | 2.4541 | 3.3226 | 0.0146 | 67.4 | 125.7 | 894.6 | 231.5 |
| 432 |  | 116.05990 | 25.98133 | 6.7315 | 21.0491 | 20.1676 | 24.1578 | 2.5844 | 3.8747 | 0.0226 | 84.9 | 121.3 | 1150.6 | 187.6 |
| 433 |  | 116.05367 | 25.97642 | 3.4825 | 15.6904 | 3.2950 | 56.7041 | 1.9126 | 8.3591 | 0.0298 | 79.5 | 119.3 | 1047.8 | 314.6 |
| 434 |  | 116.05510 | 25.97725 | 6.1817 | 16.4743 | 1.8851 | 12.8901 | 1.6719 | 3.7182 | 0.0173 | 90.6 | 119.5 | 1108 | 358.4 |
| 435 |  | 116.05604 | 25.97748 | 6.0398 | 16.8333 | 4.8899 | 11.8389 | 1.6657 | 3.7728 | 0.1700 | 79.9 | 117.9 | 1088.1 | 187.3 |
| 436 |  | 116.05821 | 25.97806 | 7.3712 | 18.6564 | 6.4850 | 11.7617 | 2.1157 | 3.6500 | 0.0175 | 71.5 | 99.8 | 776.5 | 185.4 |
| 437 |  | 116.06183 | 25.97930 | 3.5396 | 19.3765 | 6.0602 | 13.9508 | 2.3929 | 3.2007 | 0.0238 | 87.5 | 226.5 | 1135.5 | 285 |
| 438 |  | 116.01824 | 25.96357 | 5.5584 | 20.9279 | 9.0734 | 17.0270 | 1.9663 | 3.4779 | 0.0155 | 86.2 | 112.3 | 1220.6 | 237.7 |
| 439 |  | 116.01903 | 25.96343 | 12.4207 | 17.8094 | 2.3361 | 12.8487 | 2.3316 | 3.2209 | 0.0213 | 74.4 | 111.9 | 1016.1 | 184.7 |
| 440 |  | 116.01946 | 25.96355 | 5.3909 | 18.8998 | 7.8437 | 16.7552 | 1.9499 | 4.4555 | 0.0197 | 85.9 | 155.4 | 1394 | 173.7 |
| 441 |  | 116.01223 | 25.95025 | 11.8384 | 17.6764 | 4.2789 | 13.3400 | 2.3486 | 4.1019 | 0.0189 | 84.3 | 95.4 | 1131.1 | 241.7 |
| 442 |  | 116.01838 | 25.95619 | 4.4939 | 19.9013 | 6.3940 | 13.1422 | 2.8612 | 4.2955 | 0.0220 | 82.1 | 100.2 | 892.7 | 128.7 |
| 443 |  | 116.01096 | 25.94987 | 6.5153 | 19.7171 | 4.0663 | 15.3345 | 2.5307 | 4.4062 | 0.0207 | 73.9 | 112.1 | 978.3 | 200.2 |
| 444 |  | 115.99791 | 25.93349 | 6.5721 | 17.2902 | 4.1024 | 14.2879 | 2.5201 | 3.8988 | 0.0208 | 82.9 | 116.6 | 1037.4 | 218.7 |
| 445 |  | 115.99829 | 25.93353 | 5.0478 | 16.3727 | 4.0728 | 13.9773 | 2.5210 | 3.6883 | 0.0204 | 93.7 | 125.5 | 1052.9 | 228.9 |
| 446 |  | 115.99918 | 25.93347 | 7.8080 | 15.5610 | 4.0880 | 13.7109 | 2.5180 | 3.3459 | 0.0192 | 101.8 | 128.8 | 1164.3 | 227 |
| 447 |  | 116.00044 | 25.93348 | 7.0374 | 17.3763 | 3.6388 | 15.3893 | 2.4900 | 3.6299 | 0.0184 | 84 | 118.6 | 1093.7 | 218.9 |
| 448 |  | 116.00095 | 25.93320 | 6.9507 | 20.5126 | 3.0835 | 16.5422 | 2.4274 | 3.7763 | 0.0183 | 78.3 | 101.9 | 1087.7 | 202 |
| 449 |  | 116.00079 | 25.93254 | 5.1654 | 18.4152 | 9.6964 | 15.7959 | 2.4893 | 4.4791 | 0.0150 | 81.8 | 131.1 | 989.9 | 242.3 |
| 450 |  | 116.00186 | 25.93388 | 7.6286 | 21.1308 | 3.5135 | 14.8523 | 2.9252 | 3.9658 | 0.0182 | 103.6 | 122.7 | 1163.1 | 201.5 |
| 451 |  | 116.00070 | 25.93433 | 4.9705 | 18.4803 | 3.2488 | 18.5502 | 2.6641 | 3.0630 | 0.0229 | 88.5 | 123.8 | 996.9 | 338.8 |
| 452 |  | 116.00224 | 25.93499 | 7.2979 | 18.6967 | 6.0437 | 15.5469 | 2.0880 | 3.4452 | 0.0123 | 73.6 | 86.8 | 980.7 | 265.8 |
| 453 |  | 116.00166 | 25.93528 | 3.1201 | 16.1532 | 3.9309 | 15.1787 | 2.4647 | 3.0138 | 0.0278 | 80.9 | 113.6 | 983 | 305.5 |
| 454 | Huichang County | 115.76916 | 25.82620 | 6.9278 | 16.0957 | 5.9067 | 16.5277 | 3.1085 | 3.5011 | 0.0272 | 100 | 140.9 | 1218.5 | 206.8 |
| 455 |  | 115.78169 | 25.59172 | 2.9869 | 19.7460 | 2.7774 | 16.2289 | 2.7541 | 3.6622 | 0.0392 | 86.3 | 121 | 1051.5 | 231.4 |
| 456 |  | 115.61382 | 25.49320 | 8.9101 | 17.9462 | 4.1886 | 17.0610 | 1.8220 | 3.4235 | 0.0125 | 76.8 | 127.2 | 888 | 175.3 |
| 457 |  | 115.74095 | 25.38063 | 4.1893 | 18.0711 | 8.8815 | 13.6132 | 2.4272 | 3.1535 | 0.0193 | 93.6 | 127.6 | 963.8 | 218.3 |
| 458 |  | 115.74788 | 25.24164 | 1.3262 | 16.9425 | 12.3493 | 14.8079 | 2.3295 | 4.4590 | 0.0292 | 98.2 | 168 | 1191.4 | 259.8 |
| 459 |  | 115.77996 | 25.16738 | 2.7938 | 25.0925 | 6.4885 | 12.9096 | 2.0134 | 3.7721 | 0.0437 | 100.9 | 150.9 | 1172.2 | 230.8 |
| 460 |  | 115.74599 | 25.24013 | 4.7294 | 16.9780 | 27.4514 | 25.6004 | 2.0857 | 3.7865 | 0.0212 | 109.7 | 151.6 | 1188 | 229.1 |
| 461 |  | 115.74845 | 25.24374 | 2.4520 | 17.2607 | 16.0376 | 13.4596 | 2.5981 | 4.0162 | 0.0226 | 74.2 | 113.3 | 941.7 | 149.5 |
| 462 |  | 115.66404 | 25.44106 | 2.6062 | 17.5480 | 5.9454 | 12.7051 | 1.3646 | 2.9862 | 0.0114 | 75.2 | 85.1 | 840.7 | 146.1 |
| 463 |  | 115.79166 | 25.23174 | 2.0401 | 18.6538 | 8.5869 | 12.2289 | 1.9487 | 3.2296 | 0.0335 | 92.1 | 139.1 | 1155.1 | 223 |
| 464 |  | 115.72195 | 25.81950 | 2.3298 | 17.7979 | 3.9900 | 11.8489 | 2.5659 | 2.7847 | 0.0208 | 87.1 | 108.4 | 1142.8 | 247.3 |
| 465 |  | 115.71500 | 25.82551 | 4.7907 | 19.4187 | 3.2562 | 24.6238 | 1.9297 | 4.8590 | 0.0223 | 106.6 | 114.3 | 1457.7 | 216.6 |
| 466 |  | 115.72024 | 25.81388 | 3.0606 | 19.5519 | 3.4619 | 14.3432 | 1.5148 | 2.7224 | 0.0184 | 84.9 | 154.9 | 1066.9 | 287.3 |
| 467 |  | 115.70820 | 25.86486 | 2.1207 | 17.6479 | 3.5507 | 15.9382 | 3.4063 | 4.6780 | 0.0229 | 85.7 | 131.6 | 1404.7 | 243.9 |
| 468 |  | 115.55147 | 25.53839 | 2.0186 | 17.8173 | 9.4948 | 9.8979 | 1.6016 | 3.8576 | 0.0261 | 79.8 | 108.3 | 963.5 | 183.9 |
| 469 | Xunwu County  Xunwu County | 115.74636 | 25.11630 | 12.7493 | 21.1419 | 7.9709 | 14.5343 | 2.8035 | 3.9645 | 0.0203 | 88.1 | 117 | 1125.1 | 225 |
| 470 |  | 115.60969 | 24.92265 | 3.3440 | 20.8559 | 13.1361 | 12.3974 | 2.2451 | 3.8618 | 0.0162 | 97.7 | 124.8 | 1148.8 | 227 |
| 471 |  | 115.60816 | 24.92468 | 3.4289 | 20.9277 | 18.0672 | 11.4669 | 2.3559 | 3.7488 | 0.0179 | 89.3 | 135.9 | 1106.2 | 185.4 |
| 472 |  | 115.63239 | 24.97845 | 5.0416 | 16.9961 | 2.5444 | 11.4479 | 1.8052 | 2.9127 | 0.0209 | 82 | 108.3 | 1101.9 | 147 |
| 473 |  | 115.77937 | 25.11205 | 5.1542 | 19.4650 | 16.2901 | 11.0685 | 2.2349 | 4.3904 | 0.0195 | 96.9 | 86.8 | 1038 | 250.2 |
| 474 |  | 115.77542 | 25.11640 | 2.4027 | 20.1269 | 14.0604 | 13.4561 | 2.1857 | 4.7220 | 0.0182 | 95.4 | 77 | 1003.2 | 251.4 |
| 475 |  | 115.79474 | 25.11830 | 3.6691 | 18.3667 | 4.0880 | 25.6505 | 1.5318 | 4.0495 | 0.0386 | 92.1 | 124 | 1098 | 323 |
| 476 |  | 115.79563 | 25.01501 | 3.0563 | 26.7451 | 8.1248 | 11.3520 | 1.7480 | 3.9453 | 0.0166 | 84.5 | 120.6 | 944.3 | 139.5 |
| 477 |  | 115.80580 | 25.02048 | 4.3269 | 14.9111 | 5.3156 | 19.2074 | 2.2605 | 3.1511 | 0.0196 | 94.1 | 126.5 | 1120 | 298.7 |
| 478 |  | 115.66824 | 24.99718 | 5.4677 | 22.6464 | 7.2265 | 12.4499 | 2.9173 | 4.6042 | 0.0171 | 82.2 | 111.4 | 1061 | 205.9 |
| 479 |  | 115.72497 | 24.79767 | 1.9927 | 18.6958 | 2.7454 | 9.9673 | 2.7945 | 4.2413 | 0.0109 | 80.3 | 89.9 | 1145.4 | 261.1 |
| 480 |  | 115.64205 | 24.73670 | 1.4223 | 18.7350 | 4.1702 | 10.8649 | 2.7556 | 3.8852 | 0.0090 | 94.2 | 125.8 | 1248.2 | 280.7 |
| 481 |  | 115.76408 | 24.61521 | 1.4417 | 19.5100 | 3.8949 | 11.4168 | 3.5449 | 4.6346 | 0.0150 | 88.6 | 116.5 | 1305.2 | 211.5 |
| 482 | Shicheng County | 116.39499 | 26.42592 | 3.5397 | 19.6734 | 3.2853 | 16.7108 | 4.5024 | 3.5633 | 0.0346 | 92.5 | 134.1 | 1219.4 | 199.7 |
| 483 |  | 116.35215 | 26.43611 | 2.9899 | 21.9029 | 15.0170 | 16.4051 | 2.6526 | 3.8625 | 0.0272 | 112 | 138.7 | 1194.8 | 202.9 |
| 484 |  | 116.20083 | 26.11979 | 1.2339 | 18.2773 | 6.0772 | 12.2721 | 2.7118 | 4.1106 | 0.0096 | 97.8 | 157.3 | 1416 | 200.3 |
| 485 |  | 116.21854 | 26.08817 | 1.0042 | 23.3379 | 15.7935 | 10.6795 | 2.7968 | 4.1363 | 0.0339 | 80.6 | 135 | 1050 | 210.9 |
| 486 |  | 116.21988 | 26.10360 | 2.4057 | 24.1816 | 8.8916 | 16.2858 | 2.2587 | 4.0538 | 0.0576 | 91.8 | 139.4 | 1134.4 | 272.6 |
| 487 |  | 116.30949 | 26.12865 | 6.2616 | 14.2683 | 3.4684 | 14.7080 | 2.4891 | 3.5684 | 0.0229 | 94.4 | 124.8 | 1214.7 | 191.7 |
| 488 |  | 116.35793 | 26.07688 | 3.2490 | 22.3964 | 13.2428 | 12.9807 | 3.6653 | 5.7546 | 0.0222 | 92.7 | 42.5 | 1016.2 | 174.3 |
| 489 |  | 116.38464 | 26.32554 | 2.6181 | 19.7098 | 2.9638 | 10.4724 | 2.1806 | 2.9623 | 0.0109 | 81.6 | 118.6 | 1128.8 | 271.2 |
| 490 |  | 116.32312 | 26.33279 | 7.2248 | 16.2141 | 2.6738 | 12.6129 | 2.5120 | 4.0436 | 0.0285 | 86 | 112.8 | 1296.4 | 221.2 |
| 491 |  | 116.42228 | 26.49767 | 8.5489 | 18.4516 | 4.0264 | 21.9000 | 1.9943 | 3.0129 | 0.0488 | 89.9 | 114.2 | 1033.8 | 201.7 |
| 492 |  | 116.40504 | 26.47982 | 2.7654 | 17.1492 | 7.1301 | 14.8071 | 2.4148 | 3.7984 | 0.0281 | 81.9 | 107.4 | 965.7 | 126.8 |

**Supplemental Table 3** Recommended daily intake of selenium (suitable intake) and maximum tolerable daily intake of selenium for Chinese residents. The following data are from the dietary nutrient reference intakes for Chinese residents of different age groups and special groups (including pregnant women and lactating mothers) by the National Health Commission of the People′s Republic of China (2017).

| Age | Se recommended nutrient intake (RNI)/µg∙d^-1^ | Se tolerable upper intake level (UL)/µg∙d^-1^ |
| --- | --- | --- |
| 1-4 | 25 | 100 |
| 4-7 | 30 | 150 |
| 7-11 | 40 | 200 |
| 11-14 | 55 | 300 |
| 14-18 | 60 | 350 |
| >18 | 60 | 400 |
| Pregnant woman | 65 | 400 |
| Lactating mother | 78 | 400 |

**Supplemental Table 4** The distribution regularity of total Se in the navel orange fruit, pericarp, and flesh in southern Jiangxi

| Fruit parts | Total selenium content distribution | | |
| --- | --- | --- | --- |
|  | < 10 µg∙kg^-1^ | 10-20 µg∙kg^-1^ | ≥ 20 µg∙kg^-1^ |
| Fruit | 42.68% | 42.89% | 14.43% |
| Pericarp | 74.39% | 19.11% | 6.5% |
| Flesh | 92.07% | 6.50% | 1.43% |

**Supplemental Table 5** Average content of total selenium in navel orange flesh from 18 counties sampled in southern Jiangxi Province, China

| Regions | Se content (µg∙kg^-1^) | Regions | Se content (µg∙kg^-1^) | | Regions | Se content (µg∙kg^-1^) | |
| --- | --- | --- | --- | --- | --- | --- | --- |
| Yudu County | 5.71 | Shangyu County | | 5.26 | Ganxian District | | 4.17 |
| Ruijin City | 5.67 | Longnan City | | 4.81 | Xunwu County | | 4.12 |
| Ningdu County | 5.49 | Anyuan County | | 4.37 | Nankang District | | 4.01 |
| Xingguo County | 5.39 | Quanan County | | 4.34 | Shicheng County | | 3.80 |
| Xinfeng County | 5.30 | Dingnan County | | 4.30 | Dayu County | | 3.77 |
| Chongyi County | 5.28 | Zhanggong District | | 4.21 | Huichang County | | 3.55 |
