# Supplementary material for: Enrichment characteristics and dietary evaluation of selenium in navel orange fruit from the largest navel orange-producing area in China (southern Jiangxi)
Source: Front Plant Sci. 2022 Aug 8;13:881098. doi: 10.3389/fpls.2022.881098 (PMC9393740; doi:10.3389/fpls.2022.881098)

Supplemental Figure 1 The values of *RNIse* varied in different regions of the study area, including 1-4 years old (A), 4-7 years old (B), 7-11 years old (C), 11-14 years old (D), 14-18 years old (E), >18 years old (F), pregnant women (G), and lactating mothers (H).

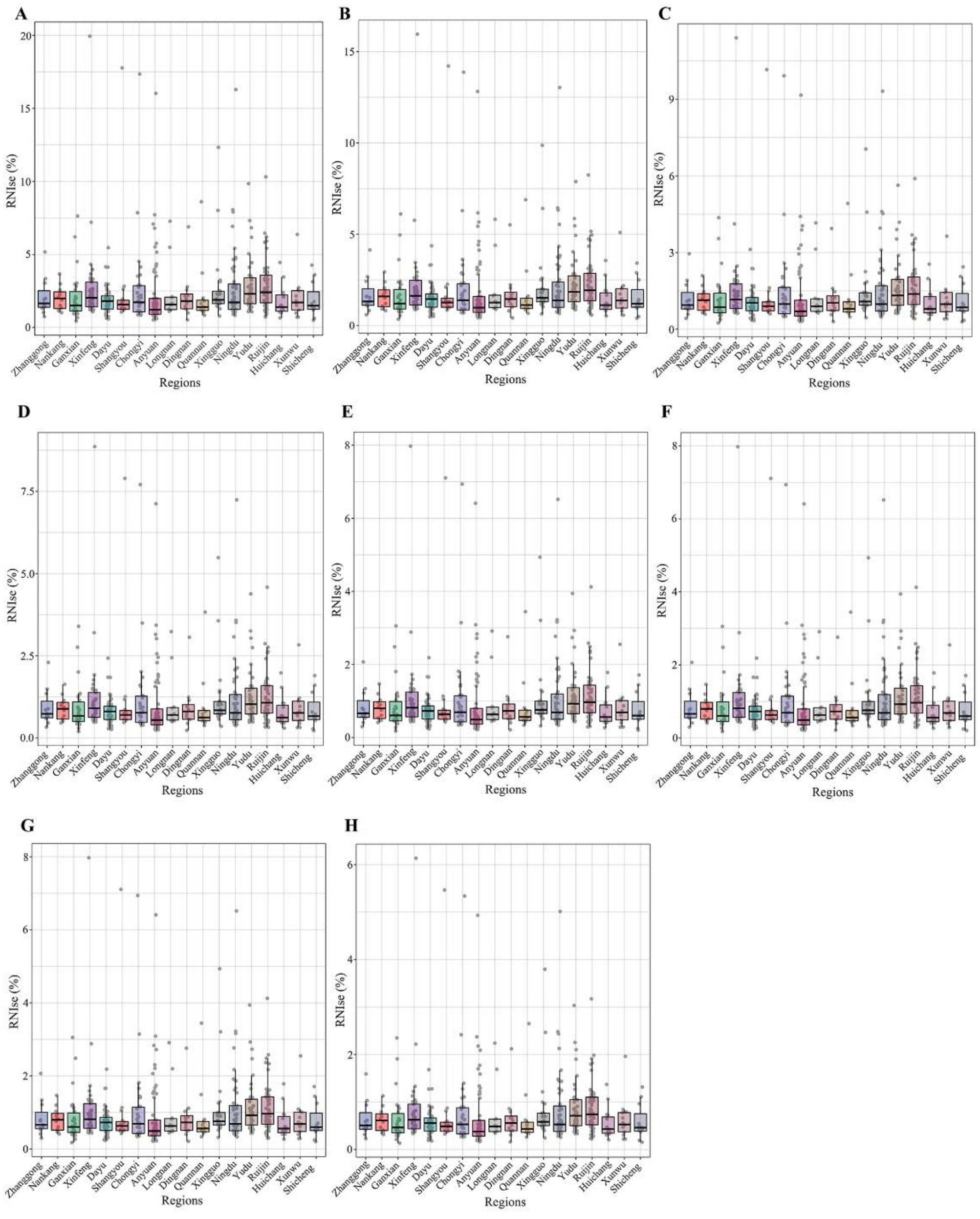

Supplement: Supplementary file 2 [file Presentation_1.pdf]
